# Additive Manufacturing for Nanogenerators: Fundamental Mechanisms, Recent Advancements, and Future Prospects

**DOI:** 10.1007/s40820-025-01874-2

**Published:** 2025-08-11

**Authors:** Zhiyu Tian, Gary Chi-Pong Tsui, Yuk-Ming Tang, Chi-Ho Wong, Chak-Yin Tang, Chi-Chiu Ko

**Affiliations:** 1 Advanced Manufacturing Technology Research Centre, Department of Industrial and Systems Engineering, The Hong Kong Polytechnic University, Hong Kong, People’s Republic of China; 2 Division of Science, Engineering and Health Studies, School of Professional Education and Executive Development, The Hong Kong Polytechnic University, Hong Kong, People’s Republic of China; 3 Department of Chemistry, City University of Hong Kong, Hong Kong, People’s Republic of China

**Keywords:** Additive manufacturing, Nanogenerators, Output performance, Energy harvesting, Self-powered sensors

## Abstract

Additive manufacturing (AM), with its high flexibility, cost-effectiveness, and customization, significantly accelerates the advancement of nanogenerators, contributing to sustainable energy solutions and the Internet of Things. In this review, an in-depth analysis of AM for piezoelectric and triboelectric nanogenerators is presented from the perspectives of fundamental mechanisms, recent advancements, and future prospects. It highlights AM-enabled advantages of versatility across materials, structural topology optimization, microstructure design, and integrated printing, which enhance critical performance indicators of nanogenerators, such as surface charge density and piezoelectric constant, thereby improving device performance compared to conventional fabrication. Common AM techniques for nanogenerators, including fused deposition modeling, direct ink writing, stereolithography, and digital light processing, are systematically examined in terms of their working principles, improved metrics (output voltage/current, power density), theoretical explanation, and application scopes. Hierarchical relationships connecting AM technologies with performance optimization and applications of nanogenerators are elucidated, providing a solid foundation for advancements in energy harvesting, self-powered sensors, wearable devices, and human–machine interaction. Furthermore, the challenges related to fabrication quality, cross-scale manufacturing, processing efficiency, and industrial deployment are critically discussed. Finally, the future prospects of AM for nanogenerators are explored, aiming to foster continuous progress and innovation in this field.

**Graphical Abstract:**

## Highlights

- The advantages of additive manufacturing for nanogenerators are firstly examined from the perspective of underlying mechanisms coupled with theoretical explanations, providing critical insights into enhancing output performance and expanding applications.
- Recent advancements in additive manufacturing for nanogenerators are systematically reviewed, emphasizing the characteristics of common technologies, their application scopes, and their impacts on nanogenerator performance metrics.
- The current challenges and future prospects of additive manufacturing for nanogenerators are explored, aiming to promote continuous advancements in this field.

## Graphical abstract

**Figure:**
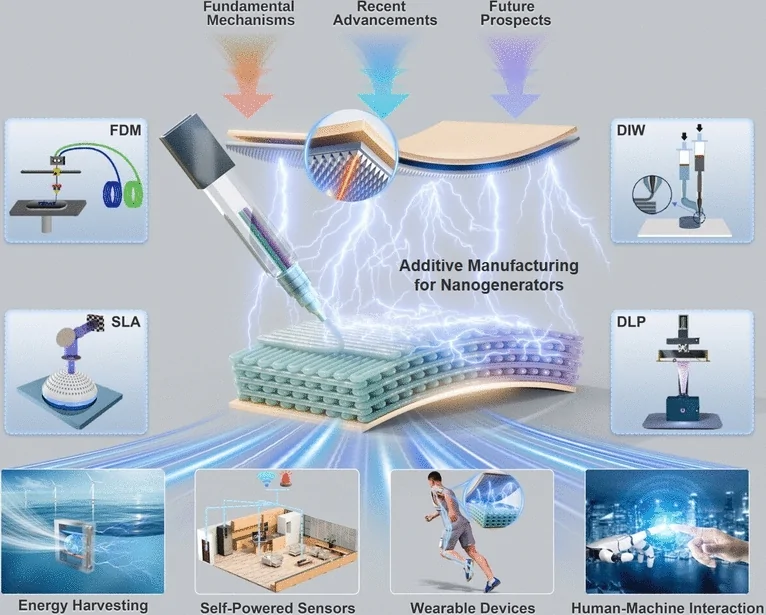

## Introduction

Traditional fossil fuels have played a pivotal role in human development; however, their extensive consumption has led to significant challenges, such as environmental pollution and the energy crisis [1, 2]. The combustion of coal, oil, and natural gas releases substantial amounts of carbon dioxide and other pollutants, exacerbating the greenhouse effect and accelerating global climate change [3]. Additionally, most traditional fossil fuels are non-renewable, and their extraction and use are associated with uneven resource distribution, energy inefficiency, and other pressing issues [4]. As fifth-generation (5G) communication technologies and the Internet of Things (IoT) advance, a vast and complex global network of billions of sensors and power sources is expected to emerge [5–7]. The large-scale, distributed, and diverse energy demand not only results in substantial energy consumption but also presents significant challenges for traditional power supply technologies, such as batteries and electrical grids, particularly regarding charging dependence, battery lifespan, and maintenance [8, 9]. Therefore, there is an urgent need to develop innovative and sustainable energy solutions. Mechanical energy represents a renewable resource, characterized by its wide availability and sustainability. Nanogenerator is an emerging, eco-friendly energy harvesting technology that convert low-frequency, distributed mechanical energy—often overlooked in both natural (e.g., wind, tides, waves) and human (e.g., motion, vibrations) environments—into electrical energy [10–13]. Nanogenerators can independently monitor specific physical or chemical properties without external power sources and transduce changes in these properties into electrical signals, thereby enabling self-powered sensing [14–16]. Among these, piezoelectric nanogenerators (PENGs) and triboelectric nanogenerators (TENGs) stand out for their high sensitivity, low cost, ease of integration, and customizability [17–20].

PENGs and TENGs typically consist of piezoelectric or triboelectric materials, electrodes, external circuits, and a supporting framework. PENGs operate based on the piezoelectric effect, in which materials with piezoelectric properties—such as inorganic materials like ZnO, BaTiO₃, and lead zirconate titanate (PZT), and organic materials like polyvinylidene fluoride (PVDF) and polyimide (PI)—generate electrical charges under mechanical stress [21–23]. TENGs operate through the coupling of contact electrification and electrostatic induction [24, 25]. When two neutral dielectric materials, such as polyimide and polymethyl methacrylate (PMMA), as well as polydimethylsiloxane (PDMS) and nylon, periodically come into contact and separate or slide against each other, alternating current is generated [26–28]. Common electrode materials for nanogenerators include metals (e.g., aluminum, copper, silver), conductive polymers (e.g., poly(3,4-ethylenedioxythiophene): polystyrene sulfonate (PEDOT:PSS)), and carbon-based materials (e.g., graphene, carbon nanotubes (CNTs)) [29–31]. Research aimed at enhancing the output performance of PENGs/TENGs and expanding their applications remains a focal point in the field. The primary strategies involve material modification, micro-/nano-surface treatment, structural optimization, and hybrid nanogenerators. However, the full potential of nanogenerators is still largely untapped due to the limitations inherent in conventional manufacturing methods. These challenges include, but are not limited to, difficulties in processing novel materials, the complexity of structural adjustments, and the inconsistency in the precise arrangement of microstructures. As a result, the limitations imposed by conventional manufacturing methods s have significantly restricted the effectiveness of the aforementioned strategies in enhancing the overall performance of nanogenerators and fully realizing their application potential. Moreover, the emerging trends of multifunctionality, miniaturized integration, and wireless portability in future-oriented nanogenerators further underscore the inadequacies of conventional manufacturing methods in meeting the evolving demands of the field [32, 33].

Additive manufacturing (AM) presents a promising and feasible approach to addressing the above issues and achieving these objectives. AM is a fabrication process that constructs objects layer-by-layer based on a digital model [34, 35]. Compared to conventional manufacturing methods like subtractive manufacturing, molding and casting, which are also commonly used for nanogenerators, AM offers inherent advantages in terms of efficiency, cost, and scalability. AM enables the direct fabrication of components with intricate geometries or complete integrated devices from digital design models, eliminating the need for time-consuming tool preparation or mold fabrication [36–38]. This capability is particularly beneficial during prototyping, where rapid design modifications and iterative optimization are often required [39, 40]. In contrast, subtractive manufacturing involves multiple processing steps, such as cutting and drilling, that are time- and labor-intensive, especially for complex structures, while frequent tool switching further undermines device precision and compromises overall fabrication efficiency. Molding and casting, although efficient for high-volume manufacturing, require the preliminary development of molds. This process incurs substantial time and cost—particularly for complex or precision designs—and limits flexibility during early-stage development. In terms of the cost, AM reduces expenses associated with tooling and molds, and its layer-by-layer approach minimizes material waste, enhancing material utilization [41, 42]. Conversely, subtractive processes consume more raw material and generate significant waste, increasing both material and labor costs [43]. While molding and casting are cost-effective for large-scale production, their advantages diminish in small-batch fabrication or prototyping—typical conditions at the current stage of nanogenerator research. Regarding the scalability, AM enables the integration of multiple materials and the direct fabrication of geometrically complex, multi-scale structures within a single process, facilitating rapid prototyping, iterative optimization, and scalable customized production [44]. Conventional methods, by contrast, require restarting the entire fabrication workflow for each design iteration, posing significant limitations to scalable development [45]. Moreover, AM offers superior sustainability and eco-friendliness, aligning well with the energy-conscious philosophy inherent in nanogenerators. Unlike conventional methods, the AM process deposits or cures material solely along the necessary printing paths defined by a digital model, significantly minimizing material waste. This process reduces raw material consumption at the source and promotes the conservation of natural resources [46]. AM enables a highly flexible and on-demand production model that accommodates the fabrication of customized or small-batch components based on real-time needs. This adaptability not only prevents overproduction but also improves material utilization. The automation and digitization of AM workflows contribute to enhanced energy efficiency, thereby reducing overall power consumption. Moreover, AM is compatible with a wide variety of sustainable materials, including renewable, recyclable, and biodegradable options, which enable material recovery and reuse, support the advancement of a circular economy, and ultimately promote long-term environmental sustainability [47, 48].

Notably, AM has achieved significant breakthroughs in addressing the critical limitations of conventional manufacturing methods, such as limited material compatibility and difficulty in fabricating complex structures, thereby offering promising solutions for the design and development of advanced devices [49–52]. Conventional techniques are typically designed to process a single type of material and often encounter significant challenges when processing materials with distinct properties, such as differences in thermal expansion coefficients or rheological behaviors, particularly in ceramics and polymer composites. In contrast, AM emerges as an advanced technique distinguished by its exceptional compatibility with multiple materials, enabling the processing of a diverse range of material systems, including metals, polymers, ceramics, and various composites [53]. For processing ceramics and polymer composites, AM enables layer-by-layer construction along with precise localized control of thermal fields and material composition, thereby enhancing design freedom and significantly reducing defect formation. For example, Hu et al. proposed a novel multi-ceramic AM technique combined with an error compensation method, which significantly enhanced both the interfacial precision and bonding among multi-ceramic components [54]. Chen et al. developed a single integrated AM technique for processing polymer composite inks to customize elastic and sustainable nanogenerators [55]. On the other hands, a single AM technique is capable of processing both ceramics and polymer composites [56–58], with only slight modifications to the processing parameters or specific components of the equipment. Moreover, conventional manufacturing methods also exhibit inherent limitations in the fabrication of complex structures in devices. The processing of high-precision structural features using conventional methods, particularly those with a resolution below 100 µm or intricate internal architectures, continues to be technically challenging and economically inefficient. The requirements for tooling, assembly procedures, and geometric constraints further impede the fabrication of ultra-precise, complex three-dimensional (3D) structures and customized components. Compared with the conventional methods, AM represents a paradigm shift by enabling layer-by-layer fabrication directly from precise digital models. This technological advancement facilitates the precise control of microscale geometries and internal architectures through high-resolution nozzles or ultra-precision laser systems. At the device level, AM is fully capable of constructing structural features ranging from a few micrometers to several tens of micrometers. In term of the highest resolution, AM can achieve nanoscale precision. For example, Huang et al. proposed an AM of high-precision ferromagnetic functional devices with fine structural features of sub-40 μm [59]. Cao et al. reported ultra-high-precision nanoscale AM of metal oxide semiconductors via multiphoton lithography, achieving a minimum critical dimension of 35 nm [60]. Additionally, advantages enabled by AM, including versatility across materials, structural topology optimization, microstructure design, and integrated printing, significantly enhance critical performance indicators of nanogenerators, such as surface charge density and piezoelectric constant. This improvement in device performance, compared to conventional manufacturing methods, will be a focal point of our discussion in the following sections. Commonly used AM techniques for nanogenerators include fused deposition modeling (FDM) [61, 62], direct ink writing (DIW) [63, 64], stereolithography (SLA) [65, 66], and digital light processing (DLP) [67, 68]. Each technique operates based on distinct principles and offers unique merits, making it suitable for fabricating and assembling various components, such as functional material layers (e.g., piezoelectric or triboelectric layers), electrodes, external circuits, and supporting and packaging frameworks in diverse PENG/TENG devices. This significantly improves the customization, precision, integration, and scalability of nanogenerators. Although previous reviews have summarized related work, most studies primarily focus on describing the progress and applications of AM-based nanogenerators, with limited in-depth analysis on how AM improves output performance [40, 69, 70]. Additionally, further discussion is needed regarding the working principles, processing characteristics, and application scopes of various AM technologies for nanogenerators, as well as guidance on selecting the appropriate techniques based on specific nanogenerator requirements.

**Fig. 1:**
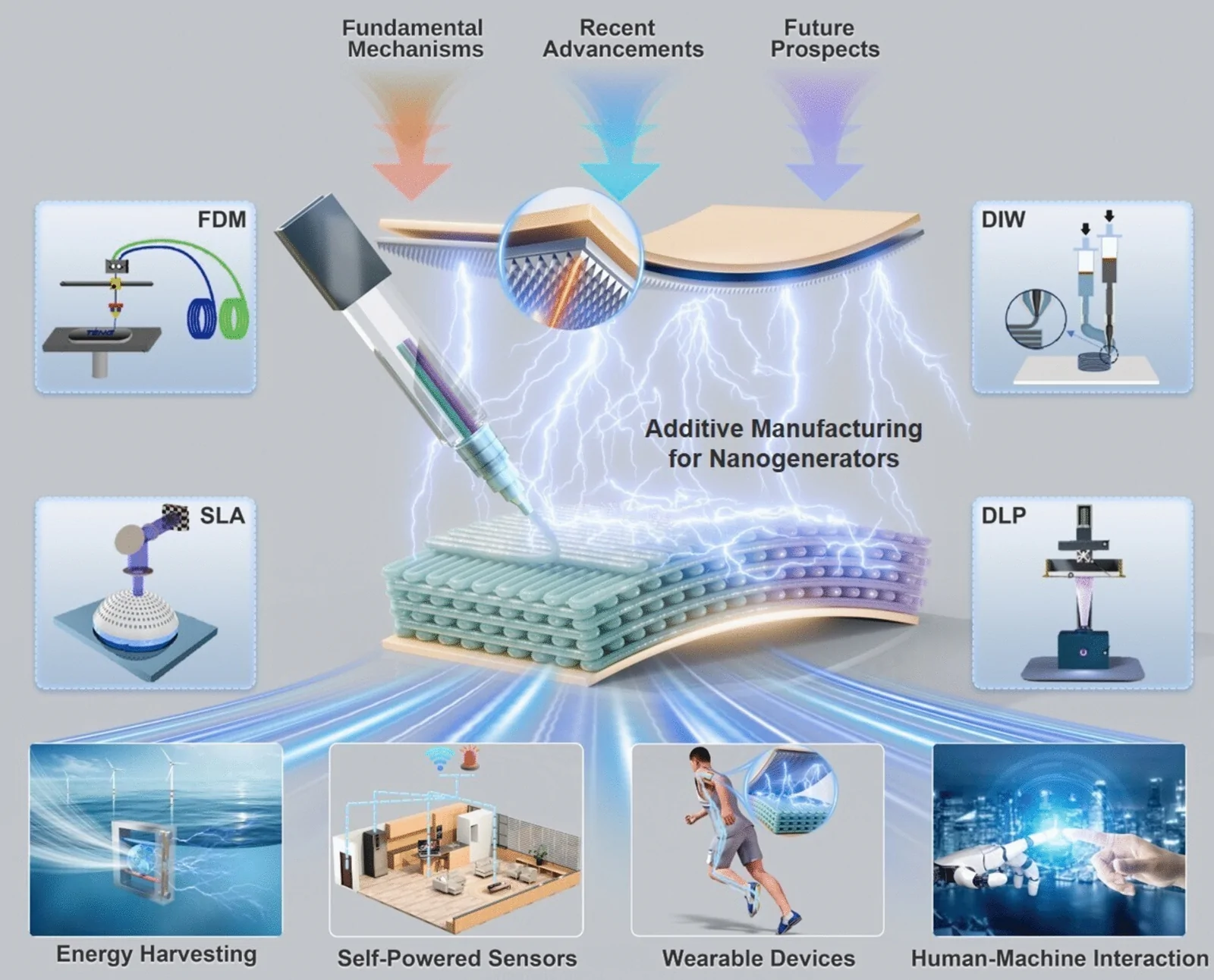
AM for TENGs/PENGs. Reproduced with permission. Reference [64] Copyright 2021, Elsevier. Reproduced with permission. Reference [66] Copyright 2020, Elsevier. Reproduced with permission. Reference [68] Copyright 2023, Elsevier. Reproduced with permission. Reference [70] Copyright 2023, John Wiley and Sons

This review aims to provide an in-depth analysis of AM for nanogenerators from the perspectives of fundamental mechanisms, recent advancements, and future prospects (Fig. 1). It intends to thoroughly present the distinct advantages of AM in enhancing the output performance and expanding the application potential of nanogenerators. Furthermore, the latest advancements in AM for TENGs/PENGs, including design strategies, fabrication methods, and applications, are to be comprehensively outlined. To establish a clear connection between AM merits and the underlying mechanisms that enhance the performance of nanogenerators, it is essential to systematically investigate commonly employed AM techniques (FDM, DIW, SLA, and DLP) in terms of their working principles, improved metrics, theoretical explanation, and application scopes. In addition, the selection of appropriate AM techniques for nanogenerators in applications such as energy harvesting, self-powered sensors, wearable devices, and human–machine interaction remains to be further explored. Furthermore, a constructive discussion of the current limitations and future prospects of AM for nanogenerators is essential to address existing challenges and foster continuous advancement and innovation in this field.

## Fundamental Principles and Performance Enhancement of Nanogenerators

### Fundamental Theories of Nanogenerators

In nanogenerators, strain-induced ions on the crystal surface generate surface polarization charges in PENGs, while the physical interaction between two materials in TENGs produces triboelectric charges on their surfaces. The classical Maxwell’s Eqs primarily describe time-varying electric fields (*E*). To account for the contribution of surface electrostatic charges and medium shape changes caused by mechanical agitation in the domain of nano-energy, an additional term *P*_S_, is introduced into the electric displacement vector *D* by Wang [71]. This term is a fundamental component of the theoretical framework for nanogenerators. Thus, the electric displacement vector can be written as [72, 73]:

1 $$D = \varepsilon_{0} E + P + P_{s}$$

Here, *Ɛ*_0_ is the vacuum permittivity. The first-term polarization vector *P* mainly arises from the external electric field, while the added term *P*_*s*_ is primarily attributed to surface charges generated by piezoelectric or triboelectric effects and the time variation in boundary shapes during mechanical stimuli. Substituting Eq. (1) into Maxwell’s Eqs, and define

2 $$D^{\prime} = \varepsilon_{0} E + P$$

From Eqs. (1) and (2), the total Maxwell’s displacement current for the nanogenerator is given by [74]:

3 $$J_{D} = \frac{\partial D}{{\partial t}} = \varepsilon \frac{\partial E}{{\partial t}} + \frac{{\partial P_{s} }}{\partial t}$$

**Fig. 2:**
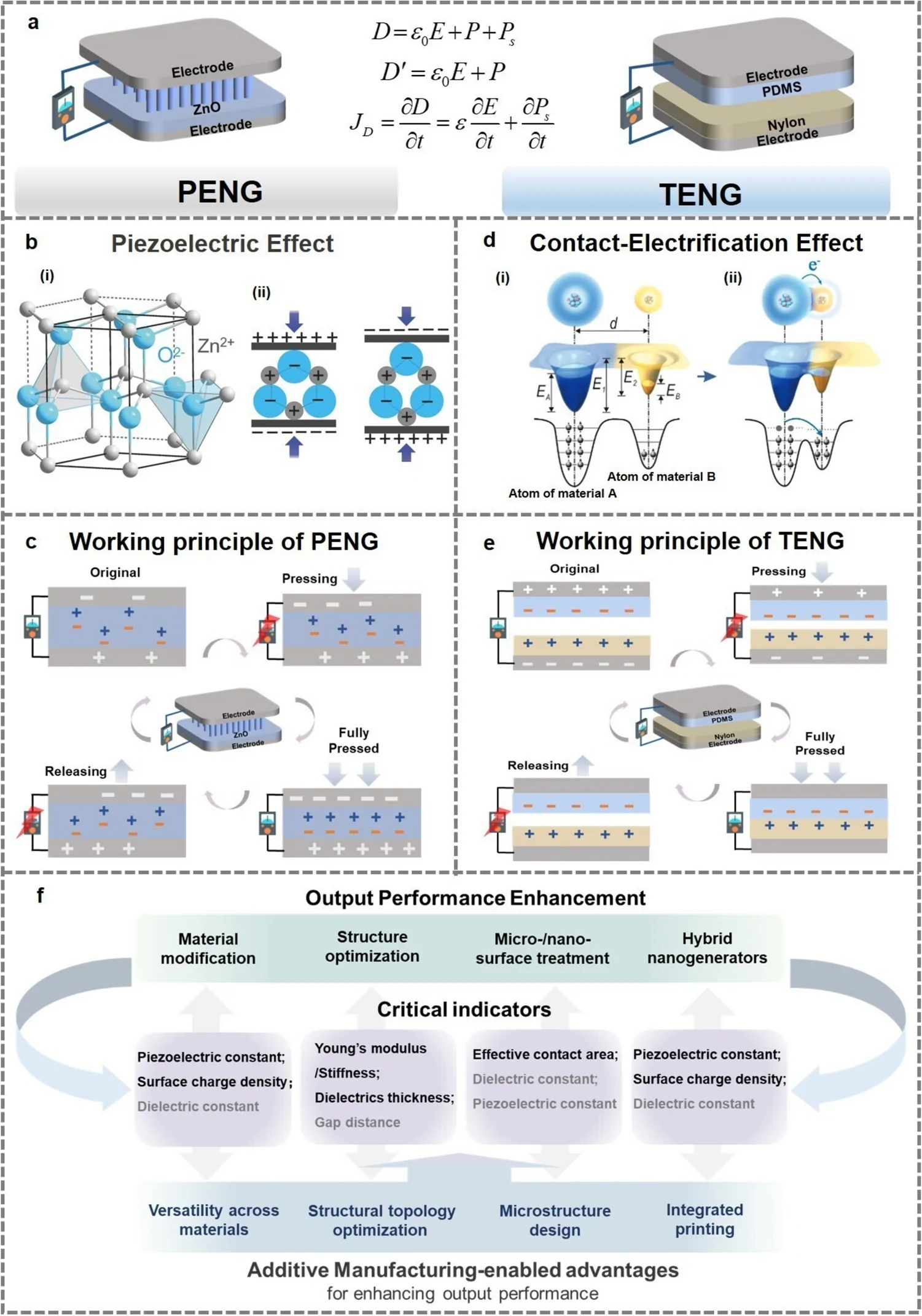
Fundamental principles and performance enhancement of nanogenerators. **a** Fundamental theory of nanogenerators. **b** Reference [79] Copyright 2016, John Wiley and Sons. (i) Atomic model of the wurtzite-structured ZnO. (ii) Piezoelectric properties and the different piezopotential in tension and compression modes of the material. **c** Working principle of the PENG during a single press-and-release cycle. **d** Reference [87] Copyright 2018, John Wiley and Sons. Schematic of the electron cloud and potential energy profile of two atoms belonging to two materials A and B, respectively, when they are: (i) before contact, (ii) in contact. **e** Working principle of the TENG during a single press-and-release cycle. **f** AM-enabled advantages for enhancing the output performance of nanogenerators

Here, the first term denotes the displacement current arising from the time-varying electric field, whereas the second term accounts for the displacement current generated by external strain fields. This establishes the theoretical foundation and origin of nanogenerators, making a significant contribution to the advancement of the nano-energy field (Fig. 2a). The following provides a detailed explanation of the operational mechanisms of PENGs and TENGs, respectively, including theoretical models, working principles, and critical performance indicators.

#### Operational Mechanisms of PENG

The concept of the PENG was first introduced in 2006, and it operates based on the piezoelectric effect [75, 76]. The piezoelectric effect refers to the generation of electric charge on the surface of specific materials under mechanical stress, such as compression, tension, or bending. Piezoelectric materials generally possess a non-centrosymmetric crystal structure, which enables the displacement of positive and negative charges under applied mechanical stress, thereby generating an electric potential [77, 78]. The wurtzite-structured ZnO crystal serves as a representative example, where tetrahedrally coordinated Zn^2^⁺ and O^2^⁻ ions are stacked along the c-axis (Fig. 2b(i)) [79]. Under equilibrium conditions, the charge centers of the cations and anions coincide are coincident. Upon the application of external stimuli, the crystal structure undergoes deformation, resulting in a separation between the charge centers of the cations and anions. This separation induces the formation of an electric dipole (Fig. 2b(ii)), which in turn generates a piezoelectric potential. A PENG typically consists of a piezoelectric layer, electrodes, and an external circuit, placed on a supporting framework or substrate. The complete working principle of the PENG during a single press-and-release cycle is shown in Fig. 2c. In the original and undisturbed state, the charges within the piezoelectric layer remain balanced, and no polarization is generated [80]. When external pressure is applied, the PENG undergoes negative strain and volume reduction, disrupting the charge equilibrium and altering the electric dipole moment, thereby inducing a potential difference between the top and bottom electrodes [81]. When connected to an external load, free electrons are driven to flow through the external circuit, partially screening the piezoelectric potential. The polarization density of the PENG reaches its highest value under full compression. As the external pressure is gradually released, electrons flow in the reverse direction within the external circuit to reestablish charge equilibrium. Given that the external stress is applied periodically, the charge distribution and electron flow fluctuate cyclically, resulting in a stable pulsed current output [82, 83]. According to the fundamental mechanism and working principles, the electrical output performance of PENGs is generally governed by multiple critical indicators, including the piezoelectric coefficient, elastic/bending moduli, and dielectric constant. While certain properties of the piezoelectric material in a PENG are intrinsic, the overall physical behavior of the integrated device can be modulated through structural or compositional modifications. The capability of a PENG to convert mechanical energy into electrical energy can be evaluated by the energy conversion coefficient (*k*) expressed as [84]:

4 $$k^{2} \sim \frac{{Yd^{2} }}{\varepsilon }$$

Here, *Y* is Young’s modulus, *d* is the piezoelectric constant, and *ε* is the dielectric constant. The comprehensive study conducted by Zou et al. [84], which systematically presented a statistical analysis of the properties of piezoelectric materials, demonstrated that materials used in PENGs typically exhibited high piezoelectric constants, thereby significantly enhancing energy conversion efficiency. During mechanical energy harvesting, the conversion performance is largely influenced by the Young’s modulus of the material or the stiffness at the device level. These critical indicators provide important guidance for fully leveraging the advantages of AM to enhance the output performance of PENGs.

#### Operational Mechanisms of TENG

The concept of TENG was first proposed in 2012, based on the coupling effects of contact electrification and electrostatic induction [85, 86]. The contact electrification effect can be explained by an overlapped electron cloud model [25]. Wang et al. used Kelvin Probe Force Microscopy (KPFM) to experimentally investigate electron transfer phenomena and proposed that such transfer occurred only when the interatomic distance between two materials was forcibly reduced below the typical bond length [72], approximately 0.15 to 0.2 nm. This condition is achieved through the application of external force, which leads to intimate atomic-scale proximity of the materials. Consequently, contact electrification can be redefined as a quantum mechanical electron transfer process that occurs across diverse materials and physical states (solid, liquid, and gas), operates within a wide range of application environments, and remains effective over a broad temperature range extending up to approximately 400 °C [24]. Specifically, as depicted in Fig. 2d(i), atoms A and B originate from two distinct materials. Each atom comprises a nucleus of positively charged protons and neutral neutrons surrounded by a negatively charged electron cloud. Prior to contact, the electron clouds remain separated without overlap. Upon application of an external force, the materials are brought into closer proximity, resulting in the initial overlap of the electron clouds. With further increase of the external force, the electron cloud overlap intensifies, leading to a transition from a single potential well to an asymmetric double-well potential and a consequent reduction of barriers [87, 88]. This process generates an energy gradient, enabling electrons to transfer from the higher-energy region to the lower-energy region, thus inducing contact electrification, as illustrated in Fig. 2d(ii). A TENG primarily consists of tribo-layers, electrodes, and an external circuit, all attached to a supporting framework. The complete working principle of the TENG during a single press-and-release cycle is shown in Fig. 2e. When the TENG is subjected to external stimuli, two dielectric materials as tribo-layers come into contact, generating equal and opposite triboelectric charges on their surfaces due to contact electrification. Upon separation of the dielectric materials, the triboelectric charges induce opposite charges on the back electrodes due to electrostatic induction, creating a potential difference between the electrodes. Connecting the electrodes with a conductive wire allows electron flow through the circuit, generating an electric current [89]. If the external mechanical agitations are periodic, the TENG generates a corresponding alternating current in response to the cyclic stimuli. Analytically, during the conversion of mechanical energy into electrical energy, the drop between the upper and lower electrodes in a typical contact-separation mode TENG is expressed as [71]:

5 $$V = \sigma_{1} (z,t)\left[ {d_{1} /\varepsilon_{1} + d_{2} /\varepsilon_{2} } \right] + z\left[ {\sigma_{1} (z,t) - \sigma_{c} } \right]/\varepsilon_{0} .$$

Here, σ_c_ denotes the surface charge density, σ_1_(*z*,*t*) represents the accumulation of free electrons in the electrodes, which is a function of the gap distance *z*(t) between the two dielectric materials. *ε*_1_ and *ε*_2_ are the dielectric constants, and *d*_1_ and *d*_2_ are the thicknesses of the two materials, respectively. *ε*_0_ is the vacuum permittivity. Under short-circuit conditions (*V* = 0):

6 $$\sigma_{1} \left( {z,t} \right) = \frac{{z\sigma_{c} }}{{d_{1} \varepsilon_{0} /\varepsilon_{1} + d_{2} \varepsilon_{0} /\varepsilon_{2} + z}}$$

From Eq. (3), the corresponding displacement current density is:

7 $$J_{D} = \frac{{\partial D_{z} }}{\partial t} = \frac{{\partial \sigma_{1} (z,t)}}{\partial t} = \sigma_{c} \frac{dz}{{dt}}\frac{{d_{1} \varepsilon_{0} /\varepsilon_{1} + d_{2} \varepsilon_{0} /\varepsilon_{2} }}{{\left[ {d_{1} \varepsilon_{0} /\varepsilon_{1} + d_{2} \varepsilon_{0} /\varepsilon_{2} + z} \right]^{2} }} + \frac{{d\sigma_{c} }}{dt}\frac{z}{{d_{1} \varepsilon_{0} /\varepsilon_{1} + d_{2} \varepsilon_{0} /\varepsilon_{2} + z}}$$

Therefore, it is noted that these critical indicators such as surface charge density, dielectric layer thickness, dielectric constant, and the interlayer gap distance significantly influence the output performance of TENGs. These findings offer viable strategies for future performance enhancement and device optimization.

### Strategies for Enhancing Output Performance of Nanogenerators

Although the theoretical foundations and operational mechanisms of nanogenerators have been extensively studied, and the field has become an interdisciplinary domain incorporating diverse technologies, their practical applications are still constrained by limitations in output performance [90, 91]. Several strategies have been proposed to enhance the output performance of the nanogenerators, broadly classified into four categories: material modification, micro-/nano-surface treatment, structural optimization, and hybrid nanogenerators.

#### Material Modification

Material modification and the development of novel materials represent the most fundamental and straightforward approaches to enhancing the output performance of nanogenerators [92–94]. For PENGs, employing materials with high piezoelectric coefficients—such as PZT, ZnO, and PVDF—or doping them with elements like lithium or yttrium can significantly enhance their piezoelectric properties [95–97]. These modifications improve charge distribution under applied forces, thereby enhancing piezoelectric output. For example, Lee et al. developed a PENG based on a grafted poly(t-butyl acrylate) (PtBA) and PVDF [98]. The copolymer, primarily consisting of the α-phase, incorporated ester functional groups from PtBA with π bonds and polar characteristics, which increased the dipole moment and nearly doubled the dielectric constant, resulting in a 20-fold enhancement in output power compared to the original PVDF-based PENG. For TENGs, choosing dielectric materials that are far apart in the triboelectric series [99], or developing dielectric materials with superior triboelectric properties, can substantially improve their output performance [100–102]. For example, a ferromagnetic-assisted Maxwell’s displacement current mechanism was proposed based on a PDMS/iron composite film. This insightful study introduced the magnetization current density (*J*_m_) into the theoretical model, thereby enhancing the displacement current and resulting in an extended form of Eq. (3) ($J_{d} = \varepsilon \frac{\partial E}{{\partial t}} + \frac{{\partial P_{s} }}{\partial t} + J_{m}$) [103]. The final power density increased by 8200% compared to a TENG composed solely of polymer materials. Additionally, the incorporation of ferromagnetic-assisted dual triboelectric layer synergy enhanced surface charge transfer and suppressed charge recombination [104], enabling the proposed TENG to achieve a peak power density of 15.2  W m⁻^2^ Hz⁻^1^, which represents a 3100% improvement over that of a conventional TENG. It is evident that material modification can effectively enhance the output performance of nanogenerators.

However, it is important to note that modified or novel materials are likely to exhibit uncertain structures and properties, which present significant challenges to conventional manufacturing techniques. For instance, a high Young’s modulus can restrict the elastic deformation range of a material, resulting in increased brittleness and posing challenges for conventional fabrication methods [84], particularly in the development of functional piezoelectric devices. Newly developed triboelectric materials often exhibit distinctive properties, such as high thermal sensitivity and chemical reactivity, which may lead to various uncertainties when exposed to high temperatures or conventional chemical-based processing techniques. In addition, nanogenerators typically require the integration of diverse novel materials, including conductive, insulating, and composite materials with specialized electrical properties. Conventional methods often encounter challenges in processing and integrating these diverse materials within a single manufacturing process.

#### Structure Optimization

The output performance can be improved by optimizing the structures of nanogenerators and shaping them into specific geometries [105–107]. These optimizations can increase the material’s deformation under external mechanical forces or enhances the contact area between layers, thereby enhancing its performance. Alternatively, designing the nanogenerator with a multilayer structure can induce varying responses between layers, promoting charge accumulation and transfer through interlayer coupling, thus boosting output efficiency [108, 109]. Moreover, flexible and deformable macroscopic structures enable the nanogenerator to adapt to various mechanical stresses or external deformations, thereby enhancing its output stability, which makes it particularly suitable for wearable devices and flexible electronics. For example, Zhang et al. developed a self-powered device based on a layer-by-layer assembled poly(vinylidene fluoride-co-trifluoroethylene) [P(VDF-TrFE)]/BaTiO_3_ (BTO) PENG to harvest biomechanical energy from carotid artery pulsations [110]. COMSOL simulation results demonstrated that the stress distribution within the multi-unit PENG in the layer-by-layer structure was significantly improved. The device exhibited a maximum instantaneous power density exceeding that of most other devices fabricated from the same materials.

However, optimizing and innovating the structure requires continuous iteration and adjustment to determine the optimal parameters, thus imposing higher demands on the manufacturing methods. Conventional manufacturing methods face several inherent limitations in the optimization of nanogenerator structures, such as constrained design flexibility, restricted tunability and variability of local features, low iteration efficiency, and high experimental costs. Consequently, there is an urgent need for a flexible, cost-efficient, and customizable manufacturing solution.

#### Micro-/Nano-Surface Treatment

Constructing surface morphology with micro-/nanostructures is an effective method to enhance the output performance of nanogenerators. For PENGs, the design of micro/nanostructures, such as nanowires, corrugations, or micropores, in piezoelectric layers can significantly enhance the surface area, thereby facilitating charge accumulation and resulting in a higher potential difference [111, 112]. For TENGs, constructing micro-/nanostructures, such as micropillars or micro-mesh, on the triboelectric layer surface increases the effective contact area between dielectric materials during the contact-separation process [113, 114]. This results in a higher surface charge density in the triboelectric layers and, consequently, a larger potential difference generated within the TENG. For example, Trinh et al. developed a novel high-aspect-ratio microneedle (MN) structured PDMS-based TENG (MN-TENG) [115]. The optimization of this surface morphology significantly enhances the output performance of the nanogenerator. The MN-TENG demonstrated an open-circuit voltage (*V*_OC_) of up to 102.8 V, a short-circuit current (*I*_SC_) of 43.1 µA, and a corresponding current density of 1.5 µA cm⁻^2^.

However, conventional manufacturing methods are inherently limited in their ability to produce micro- and nanostructures with high consistency and uniformity [116]. Even when this standard is achieved, the process typically requires additional techniques or specialized equipment, resulting in increased fabrication complexity and cost. Moreover, conventional methods for surface micro-/nano-processing typically involve high temperatures, acid etching, or complex chemical treatments, all of which may have detrimental effects on the materials. In contrast, AM can achieve the desired modifications under more controlled and milder conditions.

#### Hybrid Nanogenerators

Integrating different types of nanogenerators or combining them with other environmentally friendly energy harvesting technologies (e.g., solar cells) can achieve higher output power and improved energy collection efficiency through synergistic operation [117–119]. Furthermore, hybrid nanogenerators are capable of operating under various mechanical deformations, including vibration, bending, and collision, which makes them highly adaptable to more complex and varied real-world deployment scenarios. For example, Zhang et al. developed a vessel-like platform based on a bifilar-pendulum coupled hybrid nanogenerator module for wave energy harvesting [120]. The module consists of an electromagnetic generator, two PENGs, and two multilayer TENGs. The combination significantly improves the platform’s ability to capture wave energy, achieving a peak power density of up to 358.5 W m^−3^.

Notably, hybrid nanogenerators, which integrate multiple types of functional components—such as piezoelectric, triboelectric, and photovoltaic elements—demand careful consideration of additional parameters and intricate configurations in manufacturing process. This multi-component integration inherently results in a more complex structural design compared to single-function nanogenerators. Consequently, the manufacturing process must involve precise control and optimization to guarantee the accurate alignment, seamless integration, and reliable performance of the diverse materials and interfaces. Such meticulous fabrication is critical to fully realize the synergistic benefits and enhanced efficiency offered by hybrid nanogenerator systems.

## AM-Enabled Advantages for Performance Enhancement of Nanogenerators

The output performance of nanogenerators has significantly been improved through the four primary strategies discussed above. However, many of these strategies are still implemented using conventional manufacturing methods, such as molding, subtractive manufacturing, and manual cutting and assembly. These conventional techniques often undermine the effectiveness of the proposed performance enhancement strategies, thereby limiting both the output performance and potential applications of nanogenerators. The integration of AM into the development of nanogenerators offers distinct advantages, which can be summarized as four key merits, each closely aligned with the aforementioned strategies for enhancing the output performance of PENGs/TENGs.

### Versatility across Materials

One of the main advantages of AM for nanogenerators is its versatility, enabling the processing of a diverse range of materials. This versatility offers practical solutions for fabricating the advanced and novel materials with varying properties mentioned in Sect. 2.2.1 [121, 122]. With the continuous evolution of technology, AM has overcome the limitations of traditional materials. It is no longer confined to fabricating conventional materials such as polylactic acid (PLA), poly (ethylene terephthalateco-1,4-cylclohexylenedimethylene terephthalate) (PETG), and resin, but has evolved into a comprehensive fabrication technology capable of processing a wide range of materials, including polymers, ceramics, composites, conductive materials, and biomaterials. This expansion offers a diverse selection of materials for the development and performance enhancement of nanogenerators. Table 1 presents the various materials processed using AM for PENGs/TENGs. In contrast to conventional manufacturing methods, which are limited by mold designs and process constraints, AM enables the processing of complex, high-performance materials that are difficult produce with conventional techniques. This advancement provides a better platform for the development of novel materials, facilitating the optimization of their piezoelectric and triboelectric properties. Furthermore, AM allows for precise control over the deposition of materials layer-by-layer, ensuring uniform distribution and density at each layer. This approach not only increases material utilization and reduces waste, but also enables precise control over local compositions and properties, thereby enhancing the performance potential of modified or novel materials in nanogenerators.

**Table 1:** Materials processed by AM for Nanogenerators

PENG/TENG	Material type	Example materials	References
PENG	Polymer	PVDF	[123]
		P(VDF-TrFE)	[67]
		PLA	[124]
	Ceramics	Barium titanate (BT)	[125]
		BaTiO_3_	[126]
		PZT	[127]
	Composites	Boron nitride nanotubes/3-Trimethoxysilylpropyl methacrylate (BNNTs/TMSPM)	[66]
		PVDF/Tetraphenylphosphonium chloride (TPPC)	[128]
		BaTiO_3_/PDMS	[129]
	Biomaterial	Thermoplastic polyurethane (TPU)/PVDF	[130]
TENG	Polymer	Hydrogels	[131]
		Silicone	[132]
		PTFE	[133]
	Composites	Hybrid nanocomposite ink	[134]
		PEDOT:PSS/Polyethylene glycol diacrylate (PEDOT:PSS/PEGDA)	[68]
		Composite resin	[135]
	Conductive materials	Copper wire	[132]
		Carbon black	[64]
		MXene-based ink	[136]
	Biomaterial	Poly(glycerol sebacate) (PGS)	[137]

### Structural Topology Optimization

AM offers significant advantages in topology optimization for nanogenerators by providing exceptional adaptability to adjust device structures, particularly their macroscopic features such as shape, size, thickness, and spacing, typically at scales of millimeters, centimeters, and beyond. This can effectively address the limitations in flexibility and iterability associated with the structures discussed in Sect. 2.2.2, while also contributing significantly to improved scalability. In contrast to conventional manufacturing methods that rely on molds and complex tooling, AM constructs objects layer-by-layer, enabling the direct creation of intricate structures from computer-aided design (CAD) software, such as AutoCAD, SolidWorks and Cura. This process eliminates the need for molds, enabling the smooth translation of digital models into physical forms. In the development of nanogenerators, this capability allows for real-time adjustments to the shape and size of the structures, facilitating continuous iteration and refinement [138]. Such flexibility allows for the modification and fine-tuning of specific parameters without necessitating a complete redesign of the system. The structural optimization also enables the identification of an optimal configuration that maximizes the transmission of external stimuli into the device, directing them to the appropriate functional regions, thereby enhancing the overall output performance of the nanogenerator.

### Microstructure Design

The design and construction of precise microstructures represent a key application of AM, with a primary focus on enabling the fabrication of complex geometries at the microscale and even nanoscale. This approach is commonly employed in the surface texturing of nanogenerators and is occasionally applied to the construction of internal microarchitectures. It effectively overcomes the limitations of conventional techniques discussed in Sect. 2.2.3. Compared to conventional micro-/nano-fabrication techniques for PENGs/TENGs such as template replication [139], chemical modification [32], and electrospinning [140], AM exhibits distinct advantages in several key aspects. Firstly, AM enables the fabrication of highly complex and customized microstructures without the need for specialized molds or tooling. In contrast, template replication involves creating an initial template, which is then used to transfer microstructures onto nanogenerators. Chemical modification requires additional reagents and specialized equipment [141], while electrospinning relies on an external electric field to guide the formation of designed microstructures [142]. Conventional techniques are also limited by design flexibility and the complexity of achievable features. In comparison, the high flexibility and customization offered by AM make it particularly suitable for optimizing nanogenerator microstructures through rapid iteration and continuous refinement [66, 127]. Additionally, AM provides precise control over the directional arrangement of microstructures through digital design and layer-by-layer printing, optimizing stress distribution and charge accumulation in nanogenerators. By carefully controlling printing parameters, AM ensures high fidelity and excellent consistency in microstructure fabrication. In contrast, conventional techniques typically require multiple processing steps to achieve a designed microstructure with comparable precision [143], introducing potential errors at each stage that can compromise the final structure fidelity.

### Integrated Printing

AM enables the integration of diverse materials, structures, and components in a single printing process, which is particularly advantageous for developing hybrid nanogenerators that require incorporation of multiple functional materials for energy harvesting discussed in Sect. 2.2.4. For instance, AM can process different materials simultaneously by utilizing dual or multi- nozzles, which ensures the seamless integration of various materials in a single printing [64, 144]. This ability to co-print diverse materials is crucial for creating the complex multi-material structures that hybrid nanogenerators often require. Furthermore, the precise control of printing paths, layer height, and process parameters enabled by advanced AM techniques is crucial for the effective integration of multiple complex structures within these devices. Notably, AM allows for the complete integration of the various components in a single procedure, thereby eliminating the need for time-consuming post-processing or assembly steps that are typical of conventional manufacturing methods. This integrated printing approach not only simplifies the fabrication process but also enables the development of devices incorporating multiple energy harvesting mechanisms, thereby significantly enhancing the overall output performance of hybrid nanogenerators [125].

As demonstrated by the above analysis, the four key advantages of AM can effectively address the limitations identified in Sect. 2.2 and well align with the four principal strategies for enhancing the output performance of nanogenerators. From a theoretical perspective, AM exhibits a close correlation with the critical performance indicators of nanogenerators. Specifically, the material versatility of AM facilitates both the modification of existing materials and the development of novel materials for nanogenerators. This enables precise tuning of critical functional indicators, focusing on the piezoelectric constant, surface charge density, and dielectric constant of the materials used in nanogenerators. This implies an enhancement of the polarization effect of the piezoelectric or triboelectric layers at the material level, thereby improving the nanogenerator capability to convert mechanical energy into electrical energy and increasing its piezoelectric or triboelectric output. Moreover, the structural topology optimization inherent to AM facilitates flexible manipulation of the macroscopic geometric configuration of nanogenerators. These structural adjustments are strategically designed to optimize critical indicators including the modulus, thickness, and interlayer gap distance of the device. Such refinements enhance the extent of device deformation under external mechanical stimuli, optimize the stress distribution within the device, and promote the migration, accumulation, and directional transfer of charges. As a result, this leads to the generation of an increased electrostatic potential, thereby significantly enhancing the electrical output performance. Additionally, AM offers distinct advantages in the design and construction of microstructures, enabling precise modulation of the surface texturing and internal microarchitectures of dielectric layers and electrodes. These modifications primarily increase the effective contact area within nanogenerators and improve charge transfer efficiency. Other critical indicators, including the piezoelectric constants and dielectric constants, can also be influenced to a measurable extent. The micro- and nanoscale structural features constructed via AM can also induce localized deformation amplifications, which further strengthen the triboelectric or piezoelectric response of the system. Finally, the major advantage of AM in the integrated printing for nanogenerators lies in its capability to achieve the integration of multiple materials, structures, and components within a single manufacturing process. Through the design of precise printing trajectories and parameters, critical performance indicators of the integrated device, including the piezoelectric coefficient, surface charge density, and dielectric constant, can be effectively tuned and optimized for output enhancement. By effectively coupling multiple energy harvesting mechanisms, this substantially improves the overall output performance of the device. The clear connections between the AM-enabled advantages and the critical performance indicators of nanogenerators are shown in Fig. 2f. Overall, AM substantially enhances the responsiveness of nanogenerators to external mechanical stimuli by precisely modulating these critical performance indicators, thereby increasing the contribution of polarization field (*P*_s_) induced by these mechanical perturbations. For example, Study [145] utilized AM to precisely tailor the porosity and density distribution of the nanogenerator, and confirmed the enhancement of the polarization field through simulation. Study [66] employed AM to perform structural topology optimization on a planar piezoelectric thin film, thereby enhancing its deformability and significantly increasing the strain field induced by external excitation. Study [131] validated that the nanogenerator fabricated through AM with uniformly arranged microstructures on the surface exhibited an enhanced strain concentration effect, resulting in a polarization field substantially greater than that observed in the original devices. Theoretically, this amplification of the strain field corresponds to an improvement of the second term in Eq. (3), thus mechanistically indicating a significant enhancement in the output performance of the nanogenerator [146, 147].

## Common AM Technologies for Nanogenerators

High-performance nanogenerators fabricated using AM provide a solid foundation for their applications across various fields. Various AM techniques, such as FDM, DIW, SLA, and DLP, are widely used for fabricating the nanogenerators, significantly expanding the applications of PENGs/TENGs in energy harvesting, self-powered sensors, wearable devices, and human–machine interaction. Energy harvesting involves utilizing AM-based nanogenerators to collect energy from natural sources such as ocean waves and wind or to convert mechanical motion, vibrations, and pressure from the environment into electrical energy [117, 148]. The harvested energy can be used to power electronic devices. Self-powered sensors refer to AM-based nanogenerators continuously detecting specific objects or properties in the external environment [149, 150]. These devices convert fluctuations in target parameters into corresponding electrical signals without external power sources, enabling data transmission and early warnings. Wearable devices incorporate flexible AM-based nanogenerators that can be attached to various parts of the body for real-time monitoring of physiological states and motion signals, providing continuous data feedback for health monitoring and activity tracking [151, 152]. Human–machine interaction involves AM-based nanogenerators converting user inputs, such as touch, gesture, or speech, into electrical signals, facilitating intelligent perception and control, and bridging the physical and virtual worlds [153, 154]. Selecting an appropriate technique is crucial for optimizing nanogenerator performance and broadening their applications. Recent advancements in the four commonly used AM technologies for nanogenerators are comprehensively summarized below.

### FDM for Nanogenerators

The working principle of FDM involves the layer-by-layer deposition of a heated thermoplastic material, which is gradually shaped to form the desired structures or components of nanogenerators [155]. FDM typically uses thermoplastic filament materials, such as Acrylonitrile–Butadiene–Styrene (ABS), PETG and PLA, which melt at specific temperatures and are extruded through the nozzle in a viscous molten state [156]. The print head moves along a predefined path, typically controlled by G-code generated from CAD software, to deposit the material in precise horizontal or vertical patterns. The molten material quickly cools and solidifies, adhering to the printing platform or the previous layer. This process is repeated, with each new layer stacked on top of the previous one, until the entire object is completed. It is important to note that, when fabricating complex geometries for nanogenerators, such as overhanging or negatively angled components, FDM may require removable supports to maintain stability during printing. FDM has become a widely adopted technique in the development of PENGs and TENGs.

**Fig. 3:**
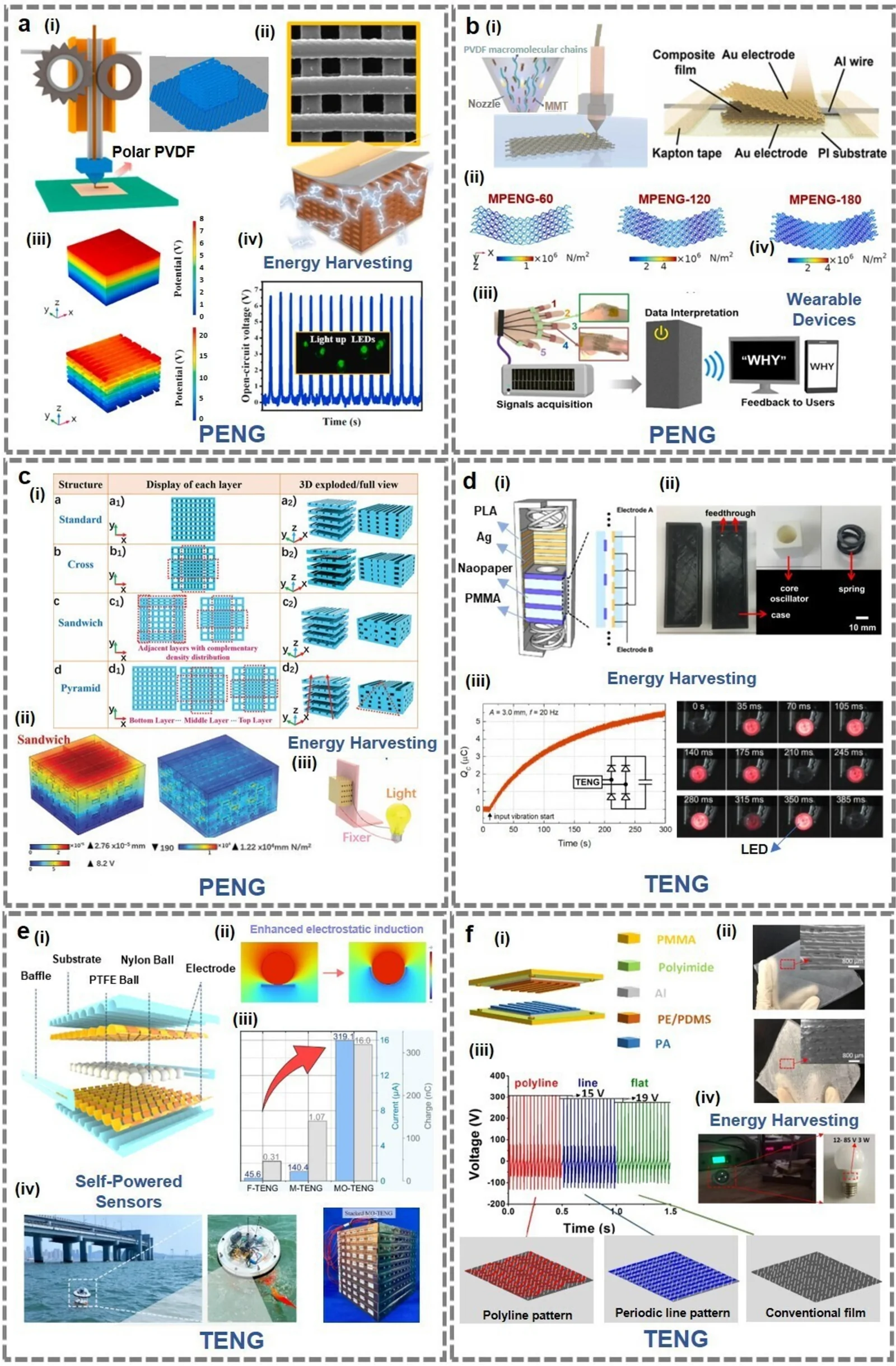
FDM for nanogenerators. **a** Reproduced with permission. Reference [128] Copyright 2021, Elsevier. (i) A depolarization-free FDM strategy for a PVDF-based nanocomposite PENG. (ii) The FDM-fabricated PENG with a complex porous structure. (iii) FEA for the porous structure and the original solid structure. (iv) PVDF-based PENG powering five commercial green LEDs. **b** Reproduced with permission. Reference [123] Copyright 2023, Elsevier. (i) FDM for a PVDF-based self-poled PENG from programmable metamaterial design. (ii) FEA for three topology-optimized structures with enhanced deformability. (iii) The FDM-fabricated PENG for motion tracking and sign language interpretation. **c** Reproduced with permission. Reference [145] Copyright 2022, John Wiley and Sons. (i) Four distinct piezoelectric structures: standard, cross, sandwich, and pyramid configurations. (ii) FEA for sandwich structures. (iii) The printed PENG for lightening optical device. **d** Reproduced with permission. Reference [158] Copyright 2018, Elsevier. (i) Structural schematic diagram of AP-TENG. (ii) FDM-fabricated components of the AP-TENG. (iii) The AP-TENG continuously illuminating the LCE light. **e** Reproduced with permission. Reference [160] Copyright 2024, Springer Nature. (i) Structural schematic diagram of MO-TENG via FDM. (ii) Simulation for the MO-TENG. (iii) Comparison of the transferred charge and current generated by the MO-TENG and conventional structures. (iv) Self-powered oceanic sensor system. **f** Reproduced with permission. Reference [161] Copyright 2018, Elsevier. (i) Schematic diagram of the FDM-based TENG. (ii) Photographs and SEM images of the tribo-layers. (iii) Comparison of *V*_OC_ of TENGs modified with different patterns. (iv) Lighting an LED bulb with the FDM-based TENG

For PENGs, the layer-by-layer fabrication of FDM enables the construction of piezoelectric layers with regularly arranged internal architectures. These structured arrangements often prompt the formation of oriented dipoles, enhancing strain coupling and optimizing potential distribution within the device. Additionally, the incorporation of structural features such as multi-layered or porous architectures lowers the stiffness of the piezoelectric material, enabling greater effective strain under identical applied pressure and thereby enhancing the resulting piezoelectric output. For example, Pei et al. introduced a novel TPPC/PVDF-based nanocomposite PENG using depolarization-free FDM (Fig. 3a(i)) [128]. The FDM-fabricated PENG exhibited a well-defined porous structure (Fig. 3a(ii)), which promoted the alignment of dipoles. Finite element analysis (FEA) showed that the porous structure substantially increased the electric potential difference between the upper and lower surfaces compared to the original solid structure (Fig. 3a(iii). For a nanocomposite containing only 5 wt% TPPC, the resulting *V*_OC_ of the PENG reached 6.62 V, approximately five times higher than that of an unmodified PVDF-based nanogenerator. This voltage was sufficient to power five commercial green light-emitting diodes (LEDs) (Fig. 3a(iv)), demonstrating the potential of FDM-fabricated piezoelectric components for energy harvesting (Fig. 3d). Moreover, the enhancement of PENG performance achieved through FDM is further attributed to topological optimization of the structure, which enables the device to overcome conventional geometric constraints, accommodate larger deformations, and thus achieve higher piezoelectric output. For instance, Pei et al. presented a PVDF-based self-poled PENG from a programmable metamaterial design using FDM technology (Fig. 3b(i)) [123]. Ion salt and montmorillonite were incorporated into the PVDF matrix to create a piezoelectric nanocomposite. Unlike conventional thin films, the FDM-constructed network structure was more prone to lateral buckling, thereby allowing for unrestricted out-of-plane twisting and bending deformations. This deformation behavior typically induced global stress softening, which enhanced the mechanical deformability of the device (Fig. 3b(ii)). Combing with simulation data, FDM facilitated the efficient determination of optimal design configurations and achieved the maximum *V*_OC_ of 9.7 V. The FDM-fabricated PENG showed significant potential for motion tracking and sign language interpretation (Fig. 3b(iii)), which underscored FDM as a powerful tool for creating wearable piezoelectric devices. Furthermore, the inherent flexibility of the FDM process enables the rapid adjustment and iterative refinement of structural parameters in topology optimization. For example, Liu et al. proposed a 3D architectural design enabled by FDM technology that allowed flexible tuning of piezoelectric output performance under identical material compositions and testing conditions [145]. Precise control of filament movement along the x-, y-, and z-axes during the printing process enabled the achievement of uniform or non-uniform distributions within the same layer and variable interlayer spacing (either tight or sparse) across different layers. This strategy facilitated the fabrication of four distinct piezoelectric structures: standard, cross, sandwich, and pyramid configurations (Fig. 3c(i)). These structural parameters were directly defined and fine-tuned during the AM process, eliminating the need for external equipment and enabling cost-effective, rapid design iterations. The sandwich structure exhibited the most optimal porosity and density distribution, offering enhanced adaptability to load transfer and stress confinement (Fig. 3c(ii)). This configuration achieved the highest piezoelectric output (8.6 V and 280 nA), which was more than twice that of the standard structure (4.2 V and 105 nA). These findings offered a powerful tool for the design and optimization of 3D PENGs, demonstrating significant potential in energy harvesting (Fig. 3c(iii)).

For TENGs, FDM allows for the precise fabrication of complex geometries, enabling the design of supporting or packaging structures for TENGs that optimize energy transfer or increase the contact area. Well-designed supporting or packaging structures can enhance the mechanical response of TENGs by maximizing the transfer of externally applied mechanical energy to the internal triboelectric layer, thereby reducing energy loss during transmission and improving overall energy harvesting efficiency [157]. Such designs also increase the contact area of the triboelectric layers, promoting greater charge accumulation and enhancing the output performance of the device. For example, Seol et al. introduced an all-printed TENG (AP-TENG) and explored design strategies for ensuring reliable operation (Fig. 3d(i)) [158]. The PVA-based structural framework fabricated via FDM consisted of two outer shells, two springs, and a core oscillator (Fig. 3d(ii)), forming a core–shell structure that effectively converted external vibrations into continuous linear sliding motion. The size of the FDM-fabricated core oscillator determined the contact force, which directly influenced the output performance of TENG. The durable AP-TENG achieved a maximum instantaneous* V*_OC_ of 98.2 V, a maximum *I*_SC_ of 13.7 μA, enabling continuous illumination of the LEDs (Fig. 3d(iii)). Additionally, the FDM-based packaging structure offers effective encapsulated protection, preventing external contaminants such as dust and moisture from infiltrating the triboelectric interfaces. This can mitigate the influence of environmental humidity on charge generation, thereby enhancing the device’s reliability under diverse conditions, including outdoor and high-humidity environments, and contributing to improved long-term output stability [159]. Besides, the encapsulation structure helps maintain the stability of the triboelectric interface by preventing misalignment or external disturbances, reducing signal fluctuations. As a result, the triboelectric layers can sustain a desired relative motion state over multiple operational cycles, enabling more stable and enhanced output performance. For example, Wang et al. reported a rolling-mode TENG (MO-TENG) by FDM for efficiently harvesting wave energy (Fig. 3e(i)) [160]. The substrate and baffles of the MO-TENG were fabricated using PLA via FDM, while the other components were encapsulated within a housing composed of two bases and baffles, forming rolling-mode TENGs (Fig. 3e(ii)). This design ensured the stable operation of the TENGs under irregular ocean waves and achieved higher output compared to conventional structures (Fig. 3e(iii)). The nanogenerator demonstrated impressive power densities, achieving instantaneous and root-mean-square values of 185.4 and 10.92 W m^−3^·Hz^−1^, respectively. By incorporating stacked MO-TENGs into a specially designed power management module, the team developed a self-powered oceanic sensor system that supported computation and remote wireless communication (Fig. 3e(iv)). This large-scale integration fully demonstrated the scalability of AM. Moreover, FDM provides a feasible and practical option for fabricating surface microstructures without the need for ultra-high resolution. Increasing the contact area between the triboelectric layers enhances the generation of opposite charges through contact electrification, thereby inducing a greater potential difference via electrostatic induction. Furthermore, well-designed surface microstructures can effectively improve the performance of TENGs by amplifying the triboelectric effect through increased frictional force aligned with external vibrations. For example, He et al. proposed a novel FDM-based strategy using polymer tubes (tubular polyethylene (PE) and nylon (PA)) as carrier boats for directly printing thermosetting materials and fabricating microstructures on the tribo-layers of TENGs (Fig. 3f(i)) [161]. The tribo-layers featuring distinct surface microstructures were illustrated in Fig. 3f(ii). To optimize the ability of the TENG to harvest vibrational energy from the ambient environment, the polymer film surface was engineered with precisely arranged patterns. Devices with polyline and periodic line patterns were fabricated and compared to conventionally processed films. The polyline-patterned device exhibited the highest output voltage, with a 34 V improvement over the conventional counterpart Fig. 3f(iii). The enhanced TENG device exhibited sufficient performance to illuminate a 3-W light bulb (Fig. 3f(iv)). Notably, the operating interface of FDM is straightforward and intuitive, requiring minimal manual configuration. This user-friendly approach facilitates the rapid transition from virtual designs to physical models, making FDM ideal for rapid prototyping, iteration, and testing. Furthermore, FDM equipment and materials are relatively inexpensive, with desktop-level FDM printers offering a cost-effective and accessible manufacturing solution for nanogenerator development. However, compared to other AM techniques, FDM typically exhibits slightly lower printing precision, which may not meet the ultra-precision requirements of certain applications. The layer-by-layer deposition process and the constraints imposed by nozzle diameter can lead to surface irregularities, which may require post-processing, such as sanding, to achieve a smooth finish.

### DIW for Nanogenerators

The working principle of DIW involves the precise, layer-by-layer deposition of gel-like materials (inks) onto a substrate to form complex 3D structures [162]. DIW typically uses high-viscosity inks, including hydrogels, polymers, ceramic slurries, metal powder suspensions, and specialized materials such as conductive or magnetic substances [125, 163]. DIW demonstrates exceptional versatility with various materials used in nanogenerators. These inks are required to exhibit suitable flow properties and viscosity to ensure uniform extrusion through the nozzle during printing. DIW utilizes pneumatic, mechanical, or electric systems to precisely control the extrusion speed and flow rate, ensuring consistent deposition onto a substrate. The nozzle movement is controlled by a computer numerical control system, enabling precise path control along the X, Y, and Z axes according to the design of the nanogenerator. Each layer of ink is extruded and partially cured by ultraviolet (UV) or heating source, while the nozzle moves along the predetermined path, depositing the next layer on top of the previous one to build the required 3D structure. DIW has been extensively utilized in the development of PENGs and TENGs.

**Fig. 4:**
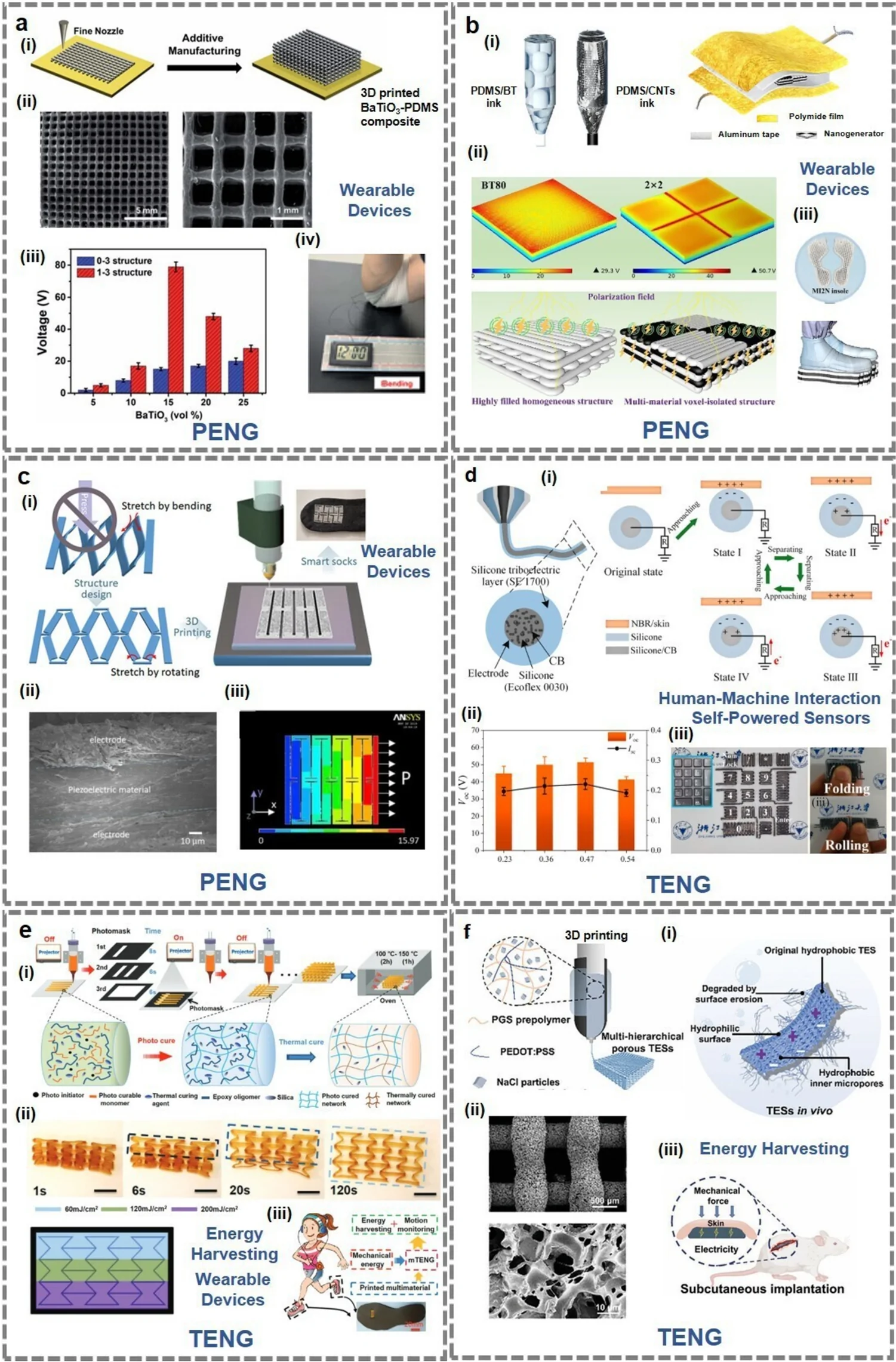
DIW for nanogenerators. **a** Reproduced with permission. Reference [129] Copyright 2021, Royal Society of Chemistry. (i) DIW for BaTiO_3_–PDMS composites. (ii) SEM images of the 3D-printed BaTiO_3_–PDMS composites. (iii) Comparison of the *V*_OC_ of two devices. (iv) An illustration of the energy harvesting of elbow movement. **b** Reproduced with permission. Reference [125] Copyright 2023, Elsevier. (i) DIW for an integrated nanogenerator with a voxel structure. (ii) FEA for the voxel-based nanogenerator. (iii) Schematic diagram and pictures of the multi-material insole. **c** Reproduced with permission. Reference [165] Copyright 2023, Elsevier. (i) DIW for a stretchable PENG with a kirigami structure. (ii) SEM image of different layers of the stretchable PENG. (iii) FEA for the T- joint-cut kirigami structure. **d** Reproduced with permission. Reference [64] Copyright 2021, Elsevier. (i) A one-pot coaxial DIW for a flexible FFTENG. (ii) *V*_OC_ and *I*_SC_ of the FFTENG with different ratios of the inner and outer diameter of the printed fibers. (iii) The FFTENGs for self-powered sensors and human–machine interaction. **e** Reproduced with permission. Reference [134] Copyright 2019, John Wiley and Sons. (i) Schematic illustration of the dynamic photomask-assisted DIW printing multi-material. (ii) Multi-material structures with controlled deformation and sequential shape memory behavior. (iii) The multi-level sequential deformable TENGs as insoles for energy harvesting and real-time monitoring. **f** Reproduced with permission. Reference [137] Copyright 2024, John Wiley and Sons. (i) DIW for a TES in tissue repair. (ii) Schematic diagram of the TES. (iii) SEM images of TES with multi-layered structure. (iv) TES for tissue-engineered cartilage in vivo

For PENGs, the precise control offered by the DIW extrusion nozzle enables the layer-by-layer construction of internally ordered structures in piezoelectric materials such as BaTiO_3_, ZnO, and PVDF-based composite inks. These ordered architectures effectively modulate stress distribution and optimize energy transfer pathways, thereby enhancing the piezoelectric output under mechanical excitation [164]. Additionally, during the DIW process, the shear stress and directional flow fields generated during the extrusion and deposition of piezoelectric materials promote the alignment of piezoelectric nanocrystals or polymer chain segments along specific orientations. This facilitates dipole alignment, increases the degree of polarization, enhances piezoelectric anisotropy, and ultimately improves the overall piezoelectric response. For example, Wang et al. proposed a BaTiO_3_–PDMS composite PENG based on DIW and dielectrophoresis for efficient electromechanical energy conversion (Fig. 4a(i)) [129]. This ordered structure facilitated the precise alignment of piezoelectric ceramic particles within the polymer matrix, thereby enhancing the piezoelectric performance (Fig. 4a(ii)). The 3D-printed dielectrophoretically aligned BaTiO_3_-PDMS composite demonstrated substantially higher output compared to composites with randomly dispersed nanoparticles (Fig. 4a(iii)), achieving a *V*_OC_ of up to 80 V and a *I*_SC_ of 25 μA. The maximum instantaneous power density reached 242 μW cm^−2^, 9 times higher than the original. This DIW-printed composite, featuring a 1–3 aligned structure, exhibited significant potential for energy harvesting and for use in wearable devices (Fig. 4a(iv)). Moreover, DIW enables spatial programmability of both material composition and structural design, allowing for the precise deposition of materials with different types or functionalities at designated locations. By controlling or switching the extrusion nozzle, the type of piezoelectric material or the filling ratio can be locally varied across different regions or layers. This spatial modulation enhances localized responses, thereby improving the overall piezoelectric output performance. Drawing inspiration from the structural design of LEGO blocks, Chen et al. utilized the DIW technique to fabricate a piezoelectric-triboelectric integrated nanogenerator with a voxel structure (Fig. 4b(i)), in which there were alternating piezoelectric and conductive inks between layers [125]. This voxel-based conductive network produced via DIW provided an optimal polarization path, ensuring more uniform polarization of the piezoelectric composite and improving polarization efficiency (Fig. 4b(ii)). A multi-material insole constructed based on this design demonstrated a *V*_OC_ of 150 V, an *I*_SC_ of 16 μA, and a short-circuit transferred charge (*Q*_SC_) of 90 nC under jumping motion. This work offered a novel strategy for designing multi-material voxel-printed flexible wearable electronics (Fig. 4b(iii)), and the LEGO-inspired architecture further highlighted the scalability of AM. Furthermore, similar to FDM, DIW can also facilitates topological optimization of PENG structures, enabling the devices to achieve greater strain by overcoming the constraints of initial geometries, thereby enhancing their piezoelectric performance. Zhou et al. developed a stretchable PENG for wearable devices based on DIW, featuring a well-designed kirigami-inspired structure (Fig. 4c(i)) [165]. The PENG, fabricated with a novel T-shaped joint cutting pattern via DIW, converted overall tilting and bending under applied stress into localized deformation at each small joint. While the main structure remained constrained within the original plane, this design significantly enhanced stretchability. As shown in Fig. 4c(ii), a distinct interface was observed between the electrode layer and the piezoelectric layer. FEA demonstrated that the T-joint structure exhibited the largest displacement (Fig. 4c(iii)), which was 6.3 times and 5.5 times greater than those of conventional kirigami and fractal-cut kirigami structures, respectively, under identical loading conditions. The proposed PENG achieved a *V*_OC_ of 6 V, an *I*_SC_ of 2  μA cm^−2^, and a maximum power density of 1.4 μW cm^−2^.

Considering the exceptional versatility across various materials, DIW technology emerges as the most effective method of multi-material integrated printing for TENGs [166]. By precisely controlling the deposition trajectories of multiple materials, DIW technology enables the creation of complex geometries with multi-material coupling, rather than merely forming structures from a single material. During the multi-material printing process, DIW facilitates bonding at both inter-material interfaces and intra-material layers. Particularly, optimizing the contact quality between the electrode and the triboelectric layer enhances the electrostatic induction effect and mitigates performance degradation due to interface delamination. For example, Wang et al. introduced a one-pot coaxial DIW technique to fabricate a fully flexible single-electrode TENG (FFTENG) with complex shapes and 3D structures (Fig. 4d(i)) [64]. This fabrication method enabled the efficient and customizable one-step printing of both the triboelectric layer and the electrode layer. More importantly, by regulating the extrusion pressures in the inner and outer channels, the ratio (*a*) between the inner and outer diameters of the printed fibers could be precisely tuned to achieve optimal output performance (*a* = 0.47), as shown in Fig. 4d(ii). The standard square FFTENG, measuring 30 mm × 30 mm, generated a *V*_OC_ of 60 V, an *I*_SC_ of 0.23 μA, a *Q*_SC_ of 58 nC, and a maximum output peak power density of 15.59 mW m^−2^. This one-pot coaxial DIW technique enabled the design and customization of various FFTENGs for self-powered sensors and human–machine interaction (Fig. 4d(iii)). Chen et al. developed a dynamic photomask-assisted DIW technique for the fabrication of multi-material TENGs (Fig. 4e(i)) [134]. Combining precise control of the DIW process with two-stage curing hybrid inks, multi-material structures with tailored tensile moduli and failure stresses were directly printed, enabling controlled deformation and sequential shape memory behavior (Fig. 4e(ii)). Owing to the differences in elastic modulus among the printed layers, the device exhibited clearly distinguishable sequential deformations under external mechanical stimuli, thereby allowing TENGs to produce multi-level responses to varying applied forces. The proposed multi-level sequential deformable TENG was further integrated into insoles for energy harvesting and real-time monitoring of motion states (Fig. 4e(iii)). Furthermore, DIW allows for the incorporation of particles such as PEDOT:PSS, CNTs, and MXene into the ink for the triboelectric layer. The precise control of the printing path ensures the uniform distribution of these particles within the designed structure, thereby offering conductive pathways that enhance electron transfer efficiency. Additionally, as previously mentioned, doping magnetic particles such as iron and nickel powders can facilitate the coupling of displacement current and magnetization current, significantly improving the output performance of TENGs. For instance, Luo et al. employed DIW technology to develop a triboelectric scaffold (TES) that integrated biomedical electrical stimulation with biomechanical motion for self-powered electrotherapy (Fig. 4f(i)) [137]. Biodegradable PGS was chosen as a matrix to resist cyclic deformation, and the TES was fabricated into a multi-layered structure with micropores by DIW to increase contact area (Fig. 4f(ii)). The PEDOT:PSS doped into the matrix functioned both as a triboelectric material and as a conductive network for electron transfer. Each moisture-resistant and hydrophobic micropore within the layered structure was designed as an independent TENG unit to enhance the overall output performance. The DIW-fabricated TES was used in bioengineering for energy harvesting to promote cartilage regeneration and repair joint defects (Fig. 4f(iii)). It is evident from the above examples that DIW is highly compatible with a wide range of materials, such as polymers, ceramics, metals, composites, biomaterials, and customized functional inks, enabling the fabrication of nearly all components of the nanogenerators, including piezoelectric and triboelectric layers, electrodes, external circuits, and supporting frameworks [135, 167]. Compared to other technologies, such as FDM and SLA, DIW typically operates at lower temperatures, offering greater material flexibility and minimizing the risk of thermal damage or deformation. Additionally, DIW can be used to fabricate nanogenerators with excellent biocompatibility, making them suitable for applications in tissue engineering, drug delivery, and artificial organs in the biomedical field. However, DIW requires inks with specific viscosity characteristics. The inks must demonstrate appropriate rheological properties and viscosity at specific temperatures to ensure uniform extrusion through the nozzle. If the ink is either too thick or too thin, it may compromise print quality and lead to nozzle clogging. Components produced by DIW generally require curing to maintain their shape such as photocuring or thermal curing, with the method depending on the ink type. This process can be time-consuming and may require additional equipment and procedures.

### SLA for Nanogenerators

SLA operates based on the principle of precise curing of liquid photopolymer material with an UV laser, constructing the 3D object in a layer-by-layer manner [168, 169]. The photopolymers used in SLA typically contain photopolymerizable monomers and initiators, which undergo a photopolymerization reaction upon UV exposure, transforming from liquid to solid. In SLA, a galvanometer mirror system directs the laser beam using electrically controlled mirrors, typically composed of aluminum or other highly reflective materials [170]. This system enables rapid and precise laser positioning along the X and Y axes, facilitating high-precision scanning based on the 3D model data of the nanogenerator components. After each layer is cured, the build platform is lowered by a fixed increment to ensure adequate exposure of the subsequent layer to UV for curing. As the platform descends, the material tank adjusts automatically, ensuring a uniform layer of liquid photopolymer covers the previously cured layer. This process is repeated until the entire 3D object is complete. Finally, the nanogenerator components fabricated by SLA typically require post-processing, including the removal of excess support structures, cleaning to remove uncured photopolymer, and further UV curing to enhance their strength and stability. Many studies on SLA for PENGs and TENGs have already been reported.

**Fig. 5:**
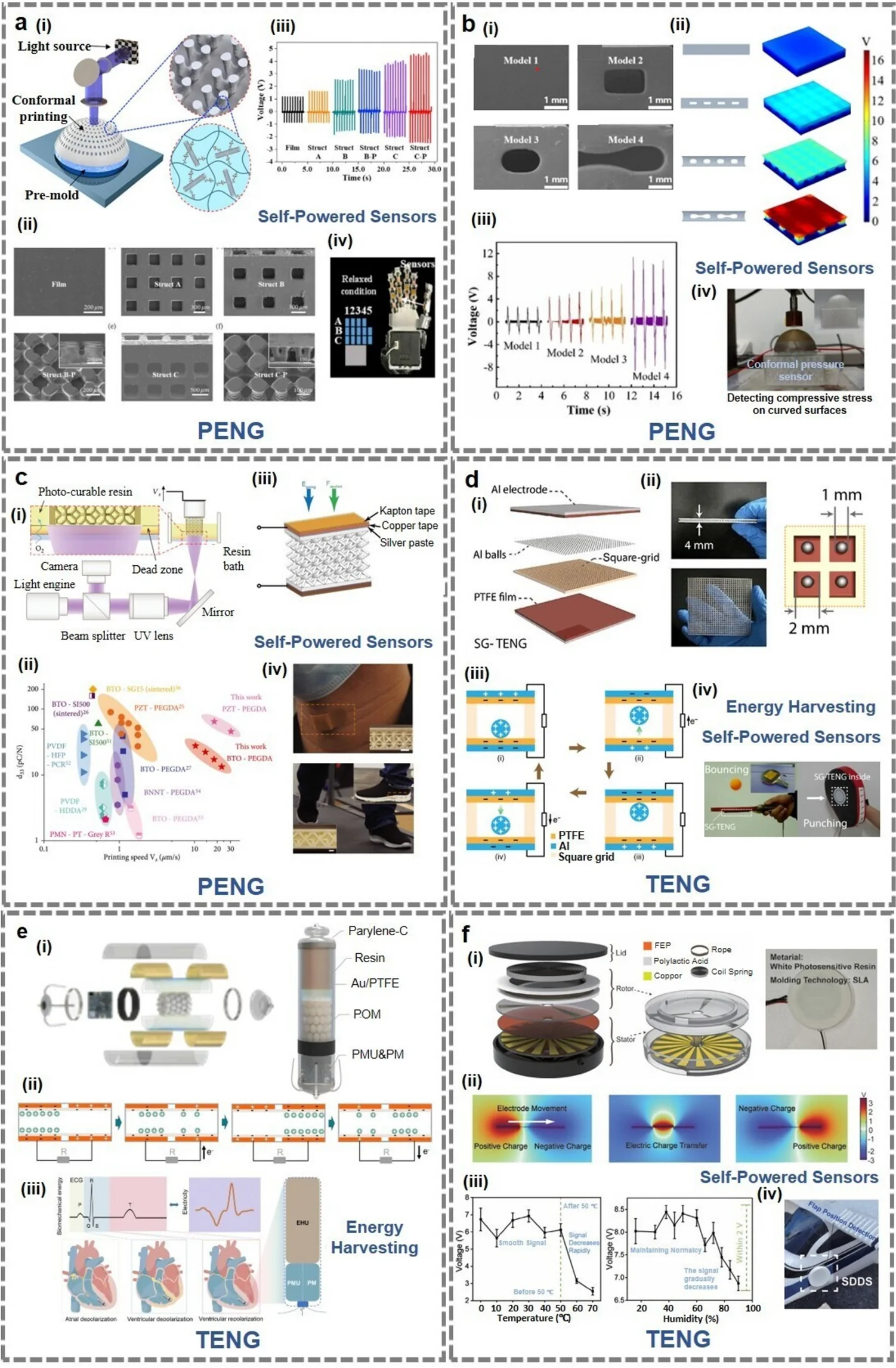
SLA for nanogenerators. **a** Reproduced with permission. Reference [66] Copyright 2020, Elsevier. (i) SLA for a BNNTs/photopolymer composite-based PENG. (ii) SEM images of different 3D-printed piezoelectric materials with various microstructures. (iii) Comparison of *V*_OC_ generated from piezoelectric materials with various microstructures. (iv) PENG for robotic tactile sensing. **b** Reproduced with permission. Reference [127] Copyright 2023, Elsevier. (i) Topological optimization model and simulation results of microstructures. (ii) SEM images of different microstructures. (iii) Comparison of *V*_OC_ generated from different microstructures. (iv) Conformal pressure sensor for detecting compressive stress on curved surfaces. **c** Reproduced with permission. Reference [172] Copyright 2022, American Association for the Advancement of Science. (i) μCLIP method beyond conventional SLA for fabrication of high-quality piezoelectric devices. (ii) Comparison between this work and other reported works in terms of printing speed and *d*_33_. (iii) The 3D-printed composite fabricated as a piezoelectric device. (iv) Piezoelectric devices for motion detection. **d** Reproduced with permission. Reference [65] Copyright 2017, Springer Nature. (i) Schematic diagram of the SG-TENG. (ii) Isolated SG-TENGs fabricated using SLA. (iii) Working principle of the SG-TENG. (iv) SG-TENGs integrated with a ping-pong paddle and boxing gloves. **e** Reproduced with permission. Reference [175] Copyright 2024, Springer Nature. (i) Schematic diagram of the SICP. (ii) Working principle of the SICP. (iii) SICP inserted into the right ventricle for implanted bioelectronics. **f** Reproduced with permission. Reference [176] Copyright 2023, John Wiley and Sons. (i) Schematic diagram of the SDDS. (ii) Working principle of the SDDS. (iii) Signal output of SDDS at different temperatures and humidity levels. (iv) Sensors are mounted on the fuselage to monitor the position of the flaps

For PENGs, SLA enables point-by-point photopolymerization with submicron spatial resolution, allowing piezoelectric materials to be fabricated into highly ordered periodic microstructures such as honeycomb lattices, pillar arrays, and gradient architectures. Such topological optimization of microstructures can significantly enhance the device’s sensitivity to external mechanical stimuli by enabling efficient stress transfer to the piezoelectric regions or by amplifying local strain concentration, thereby increasing polarization intensity and output [171]. Moreover, these structures offer additional strain pathways and deformation gradients, which facilitate higher polarization charge density. Some structure, like cavity and porous designs, provide greater deformability, improving the output performance of the piezoelectric layer under equivalent mechanical stress. In addition, by precisely controlling the light exposure path and curing patterns, SLA enables the fabrication of microstructures with anisotropic geometries—such as columnar arrays or linear bridging networks. These structures can, under certain conditions (e.g., external field assistance or flow-induced alignment), serve as physical templates that facilitate the orientation of molecular chains or nanofillers along predefined directions. This promotes oriented polarization within the piezoelectric material, thereby enhancing energy conversion efficiency. For example, Zhang et al. proposed a BNNT/photopolymer composite-based PENG with tunable interfaces and microarchitectures fabricated via SLA (Fig. 5a(i)) [66]. BNNTs (0.2 wt%) were incorporated into the photocurable polymer solution, which was then used to fabricate PENGs with customized interfaces and microstructures (Fig. 5a(ii)). The design of different structures based on topology optimization boosted the piezoelectric response (Fig. 5a(iii)), resulting in a maximum sensitivity of 24 mV kPa^−1^, which was tenfold higher than that of the flat pristine BNNT-based composite. The novel PENGs were successfully used as robotic tactile sensors to detect spatial pressure distribution on uneven surfaces (Fig. 5a(iv)). To enhance piezoelectric output, Wang et al. investigated the material system and structural design of SLA of piezoelectric composites [127]. By performing structural topology optimization on an initial model with a 100% volume fraction (Model 1), three optimized structures with reduced volume fractions of 85% (Model 2), 70% (Model 3), and 60% (Model 4) were obtained (Fig. 5b(i)). These structural adjustments required no additional molds and were directly constructed through SLA, thereby reducing costs and improving efficiency. FEA revealed that as the volume fraction decreased—that was, as less material was retained during the optimization process—the output voltage of the piezoelectric devices increased correspondingly (Fig. 5b(ii)). At lower volume fractions, the structures exhibited greater deformation, and stress was more effectively transferred within the piezoelectric framework, thereby significantly enhancing the device performance. Model 4 achieved the highest output of 10 V, representing a nearly fourfold increase over the flat solid structure in Model 1 (2.5 V) (Fig. 5b(iii)). The piezoelectric sensitivity of the topology-optimized composite reached up to 30 mV kPa^−1^. This conformal pressure sensor with fabricated by SLA effectively detected compressive stress on curved surfaces (Fig. 5b(iv)). Moreover, SLA technology enables the fabrication of functional composite materials with uniformly dispersed particles by utilizing multi-material printing or by pre-doping functional particles (such as PZT, BaTiO₃, ZnO, etc.). This approach ensures a uniform distribution of the fillers during processing, thereby preventing the formation of “inactive regions” caused by irregular dispersion. Sometimes, precise control over the process allows for localized functional enhancement, thereby improving the overall performance output. For instance, Liu et al. developed an advanced fabrication approach beyond conventional SLA to process a photocurable resin containing piezoelectric nanoparticles for the production of high-performance piezoelectric devices (Fig. 5c(i)) [172]. This approach based on continuous liquid interface production (μCLIP), facilitated the continuous printing of microscale 3D piezoelectric structures at a speed of up to ~ 60 μm s⁻^1^, which was more than ten times faster than previously reported methods (Fig. 5c(ii)). The topology-optimized 3D-printed piezoelectric devices (Fig. 5c(iii)), when fabricated with 30 wt% f-BTO, exhibited a bulk piezoelectric charge constant of 27.70 pC N⁻^1^. The printed devices demonstrated excellent mechanical flexibility and sensing performance for self-powered sensors (Fig. 5c(iv)).

For TENGs, the ultra-high resolution of SLA enables the construction of compact, geometrically complex support structures even within limited spatial constraints, making it particularly suitable for miniaturized devices with tightly coupled components. SLA further facilitates the well-encapsulated designs by enabling the fabrication of sealed microcavities or protective housings. These structures effectively shield the device from environmental factors to reduce charge neutralization and dissipation, thereby enhancing charge retention and improving the output stability of the devices [173, 174]. For instance, He et al. introduced a square grid TENG (SG-TENG) fabricated using SLA for vibrational energy harvesting and impact force sensing (Fig. 5d(i)) [65]. SLA enabled the fabrication of individually isolated TENG units within a 30 × 30 grid framework, where each cell measured only 2 mm × 2 mm (Fig. 5d(ii)). This grid configuration allowed the SG-TENG to efficiently harvest vibrational energy across a broad frequency spectrum and operate under various vibration angles (Fig. 5d(iii)). When integrated into sports equipment such as ping-pong paddles and boxing gloves, the SG-TENG generated a *V*_OC_ of 10.9 ± 0.6 V and an *I*_SC_ of 0.09 ± 0.02 μA upon impact, while also enabling real-time monitoring of punch and kick frequency and intensity (Fig. 5d(iv)). Similarly, Liu et al. developed a self-powered intracardiac pacemaker (SICP) in a swine model using SLA (Fig. 5e(i)) [175]. The SICP was fabricated into a microscale capsule structure via SLA, harvesting biomechanical energy from cardiac motion through working principle of TENG (Fig. 5e(ii)). The 3D-printed encapsulation structure ensured its stable performance in the high-humidity in vivo environment and achieved a *V*_OC_ of 21.8 V, an *I*_SC_ of 0.25 μA, and a *Q*_SC_ of 6.4 nC. This approach highlighted recent advances in self-powered medical devices and offers a potential solution to the energy limitations of implanted bioelectronics (Fig. 5e(iii)). Zhou et al. developed a lightweight self-powered digital displacement sensor (SDDS) using SLA technology (Fig. 5f(i)) [176]. The SDSS had a volume of < 11.1 cm^3^ and a weight of < 9.5 g, with all components encapsulated internally and operating based on a free-standing-mode TENG (Fig. 5f(ii)). Owing to its robust encapsulation, the device maintained stable performance under conditions of 50 °C and 100% humidity, consistently generating a *V*_OC_ > 6 V (Fig. 5f(iii)). Leveraging its excellent environmental adaptability, the SDDS was integrated on an unmanned aerial vehicle (UAV) to monitor the position of flight actuators in real time (Fig. 5f(iv)). The above examples clearly illustrate that SLA, with its high precision and resolution, is particularly well-suited for fabricating complex microstructures, finely detailed surfaces, and structures featuring hollow forms, curved geometries, multilayer designs, and internal pores. However, SLA processing for nanogenerators is typically slower, particularly when handling high-resolution and complex models, due to the extended time required for precise scanning and curing of each layer. Additionally, the cost of SLA equipment and materials is relatively high, particularly for systems requiring ultra-high precision. Furthermore, the size of the nanogenerators may be constrained by the working area of the UV laser in SLA.

### DLP for Nanogenerators

DLP utilizes a digital projector to direct light onto a liquid photosensitive material, such as photopolymer resin, which solidifies upon light exposure, enabling the gradual construction of a 3D object layer-by-layer [177, 178]. Typically, the photosensitive material is a liquid polymer that undergoes a chemical reaction when exposed to specific wavelengths of light, transitioning from liquid to solid to form a stable structure [179]. While similar to SLA in principle, DLP offers superior efficiency and precision due to its digital control of the light source. The core element of the DLP technology is a digital projector, typically consisting of a digital light source (e.g., high-power LEDs) and a digital micromirror device (DMD) [180]. The DMD consists of numerous micro-mirrors that rapidly tilt to reflect light onto designated areas. In DLP, the model of the desired nanogenerator component is sliced into thin layers based on the required resolution and design, with the pattern of each layer mapped onto the DMD panel according to the model data. The DMD projects the pattern of each layer onto the surface of the liquid photosensitive material. The intensity and direction of reflection of each pattern determine the degree of solidification in the corresponding regions of the material. Upon light exposure, the material undergoes a crosslinking reaction in the illuminated areas, forming a solid structure, while unexposed regions remain liquid. After each layer cures, the print platform slightly moves down, and the projector projects the next layer pattern, repeating the process until the entire 3D nanogenerator component is complete. DLP technology has been extensively utilized in the development of PENGs and TENGs.

**Fig. 6:**
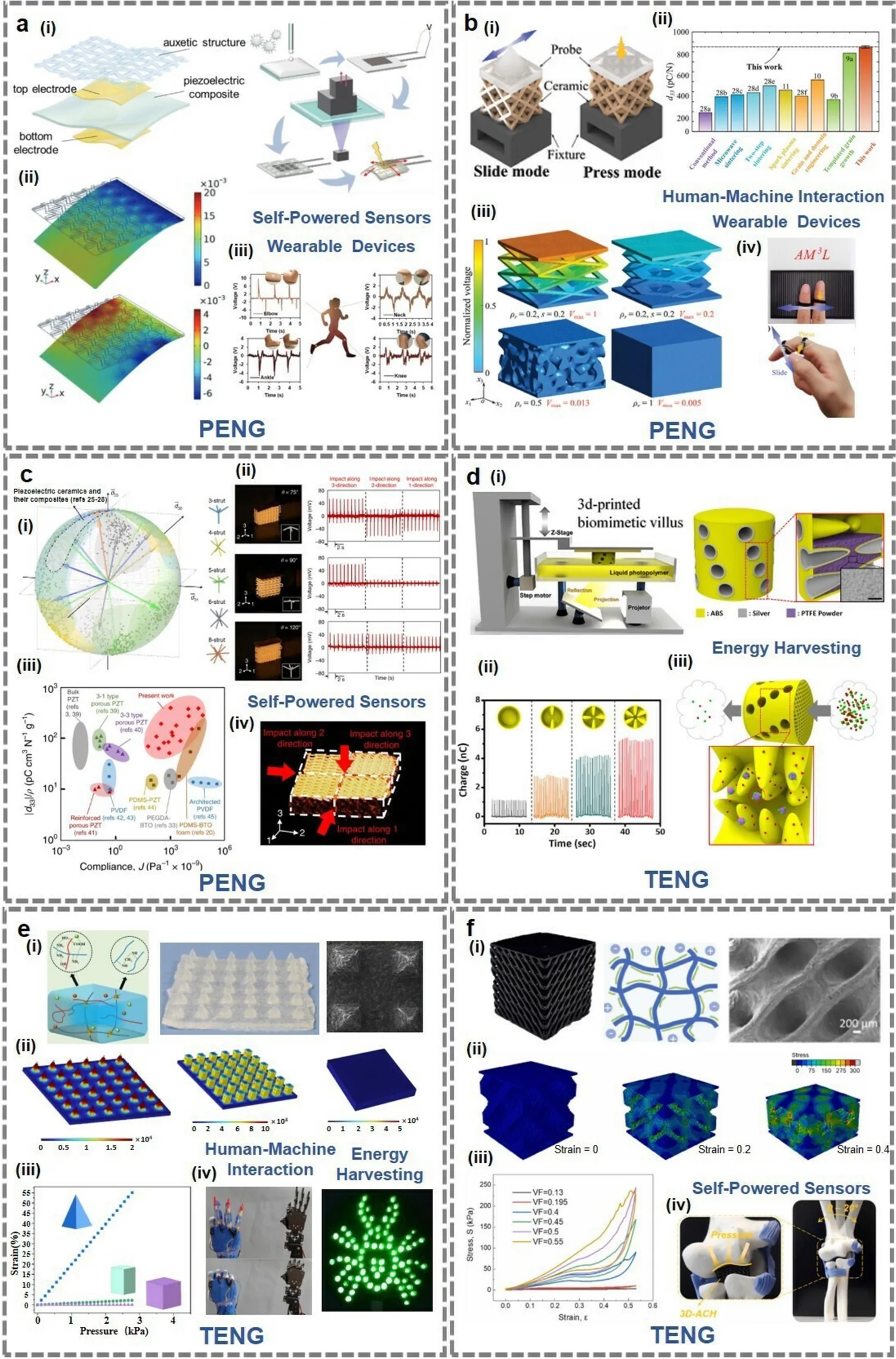
DLP for nanogenerators. **a** Reproduced with permission. Reference [67] Copyright 2023, John Wiley and Sons. (i) DLP for a thin-film PENG with the auxiliary structure. (ii) FEA for the auxiliary structures. (iii) Thin-film PENGs for self-powered and wearable devices. **b** Reproduced with permission. Reference [126] Copyright 2024, John Wiley and Sons. (i) The sliding-mode and pressing-mode devices fabricated by DLP. (ii) Comparison among the maximum *d*_33_ in this work and others studies. (iii) FEA for different structures. (iv) A game control setup and a wearable webpage interactive device. **c** Reproduced with permission. Reference [182] Copyright 2019, Springer Nature. (i) A dimensionless piezoelectric anisotropy design space accommodating different 3D node unit designs with distinct distributions. (ii) Real-time voltage outputs of 3D node units under impact coming from three directions. (iii) Comparison of specific piezoelectric charge coefficients and elastic compliance between this work and others studies. (iv) 3D digital metamaterial building blocks for force directionality sensing. **d** Reproduced with permission. Reference [133] Copyright 2025, Elsevier. (i) Schematic diagram of the BV-TENG fabricated by DLP. (ii) Comparison of *V*_OC_ generated from different microstructures. (iii) BV-TENG for dust filters. **e** Reproduced with permission. Reference [131] Copyright 2025, Elsevier. (i) PSL-TENG fabricated by DLP with deformable microstructures. (ii) FEA for three microstructures. (iii) Comparison of stress distribution of different structures. (iv) PSL-TENGs for powering 100 LEDs and for electronic skin in huma-machine interaction. **f** Reproduced with permission. Reference [68] Copyright 2023, Elsevier. (i) DLP-printed architected conductive hydrogel-based DC-TENG. (ii) FEA for the strain field in structured hydrogel. (iii) Stress–strain curves of structured hydrogels with different volume fractions. (iv) DC-TENGs with excellent alignment and adhesion on surfaces of artificial arthrosis for sensing

For PENGs, DLP enables the 3D printing of anisotropic and directionally responsive piezoelectric materials by constructing well-ordered microstructures and implementing precise topological optimization. This strategy facilitates the enhancement of piezoelectric performance by optimizing stress transmission pathways, thereby promoting localized strain amplification and polarization concentration across various directions. Specifically, transitioning the piezoelectric response mode from the conventional 3–3 mode (where the stress and polarization directions are aligned) to the 3–1 mode (where these directions are orthogonal) has been demonstrated as an effective approach to improve output performance. Mechanistically, the 3–1 mode typically induces a piezoelectric response through lateral stretching or bending, offering significantly higher localized strain compared to the 3–3 mode. This advantage is particularly evident in thin-film or fiber-based piezoelectric structures, where such deformations enhance charge migration [181]. Moreover, unlike the 3–3 mode, which depends on high compressive loads, the 3–1 mode can maintain effective piezoelectric output under low-stress conditions, making it more suitable for flexible and wearable applications aimed at harvesting small-scale mechanical energy. For example, Zhou et al. developed a film-type PENG using DLP technology, capable of operating in the 3–1 mode under bending conditions (Fig. 6a(i)) [67]. FEA revealed a co-directional elastic effect enabled by the 3D-printed auxetic structure, whereby the bending-induced energy harvesting mechanism was converted into tensile deformation across the entire piezoelectric film (Fig. 6a(ii)). This significantly enhanced the strain within the piezoelectric layer, increasing the bending *V*_OC_ of the PENG by 8.3 times, thereby overcoming the limitations of conventional bending-based energy harvesting methods in piezoelectric films. The unique auxiliary structure also provided precise control over the stretching strain without any over-stretching, serving as a bending motion sensor for human motion monitoring (Fig. 6a(iii)). Shi et al. employed DLP to fabricate ferroelectric metamaterials with complex honeycomb-like piezoelectric structures, designed for both sliding and pressing operation modes (Fig. 6b(i)) [126]. The unique integration of truss load states and polarization directions enabled a maximum *d*_33_ value exceeding that of BaTiO₃ ceramics optimized via conventional material processing and sintering techniques (Fig. 6b(ii)). Topological optimization combined with simulations of various structures showed that the octet truss achieved the highest normalized voltage, with values 5, 77, and 200 times greater than those of the tetrakaidekahedral, foam, and solid ferroelectric structures, respectively (Fig. 6b(iii)). Building upon this, a game control setup for human–machine interaction and a wearable webpage interactive device capable of simulating five keyboard functions were developed (Fig. 6b(iv)). Cui et al. developed a method for the directional design of piezoelectric materials using DLP technology and fabricated devices with enhanced piezoelectric performance that can respond to pressure from multiple directions (Fig. 6c(i)) [182]. By tailoring the projection patterns of 3D node units, the electric field was effectively manipulated, enabling directional modulation of the piezoelectric coefficient tensor. The voltage response of the activated piezoelectric metamaterials in a given mode could be selectively suppressed, reversed, or enhanced by applied stress (Fig. 6c(ii)). This approach significantly outperformed conventional piezoelectric materials in terms of charge coefficients and elastic compliance (Fig. 6c(iii)). The 3D digital metamaterial building blocks were further stacked or printed for force directionality sensing (Fig. 6c(iv)).

For TENGs, surface microstructures rapidly fabricated via DLP can substantially increase the effective contact area per unit surface. This increased contact area promotes more efficient electron transfer during contact-separation or sliding processes, thereby improving the surface charge density of the triboelectric layers. Moreover, the periodic surface or internal microstructures fabricated through DLP can induce concentrated strain and more distinct elastic deformation regions under applied force, thereby enhancing the mechanical response of the triboelectric layers. This improvement strengthens the polarization effect, leading to enhanced output performance. For instance, Yoon et al. developed a biomimetic villous structure TENG (BV-TENG) with high-resolution DLP, significantly increasing the surface area beyond traditional structural limits (Fig. 6d(i)) [133]. The test results indicated that as the number of villi increased and the surface area expanded, the output performance of the device improved accordingly (Fig. 6d(ii)). These models with varying numbers of villi were rapidly and cost-effectively fabricated using DLP. Compared to conventional planar structures, the DLP-printed BV-structure exhibited a 300% increase in the surface area, which resulted in 5 times and 4 times enhancement in power output performance along the vertical direction and rotational direction mode, respectively. This BV-TENG efficiently converted mechanical energy into triboelectricity for dust filters (Fig. 6d(iii)). Similarly, Chen et al. developed a polyacrylamide/sodium alginate/lithium chloride (PSL) hydrogel-based quadrangular pyramidal microstructure TENG (PSL-QTENG) using DLP technology (Fig. 6e(i)) [131]. To optimize the performance of the deformable microstructures, FEA was conducted to simulate the stress distribution in two distinct geometries, quadrangular pyramids and square pillars, and the results were compared with those of a flat structure (Fig. 6e(ii)). The results revealed that the quadrangular pyramid microstructure exhibited strain concentration at the pyramid tips and generated significantly higher strain than the other two structures under the same applied pressure (Fig. 6e(iii)). Under continuous impact stimulation, the PSL-QTENG achieved a *V*_OC_ of up to 201.4 V and powered 100 LEDs. Moreover, it demonstrated strong potential for application as electronic skin in human–machine interaction (Fig. 6e(iv)). Yang et al. developed a continuously DLP-printed architected conductive hydrogel-based direct current TENG (DC-TENG) (Fig. 6f(i)) [68]. The synergistic interaction between the continuously printed internal bending structures and the crosslinked polymer network significantly enhanced the compressive flexibility of the device. FEA demonstrated that, compared to bulk hydrogels, the 3D-printed structured hydrogel exhibited enhanced stress transfer and mechanical energy dissipation pathways, attributed to localized stress concentration (Fig. 6f(ii)). Moreover, DLP enabled precise control over the volumetric fraction, thereby further optimizing the strain response of the printed conductive hydrogel to external stress (Fig. 6f(iii)). The device generated DC in response to mechanical stimulation, with a *V*_OC_ of 1.15 V and an *I*_SC_ of 6.61 μA. It achieved excellent alignment and adhesion to the complex surfaces of artificial joints, enabling effective pressure and strain sensing (Fig. 6f(iv)). From the above examples, it is evident that DLP technology, with its digital projector capable of generating ultra-high-resolution images for each layer with exceptional precision, is particularly well-suited for fabricating microstructures. These microstructures help improve stress distribution and increase contact area, thereby enhancing the output performance of nanogenerators. Additionally, DLP uses a digital light projector that projects the pattern of an entire layer onto the resin surface, significantly increasing printing speeds compared to point-by-point curing methods like SLA. This advantage is particularly pronounced when fabricating small objects or multiple sophisticated components, where DLP substantially improves the production efficiency. However, unlike FDM and other AM technologies, DLP encounters difficulties in fabricating large-sized nanogenerators due to constraints in the projector light source and projection area, often requiring components to be divided into multiple parts and increasing post-processing and assembly efforts.

## Hierarchical Relationships Between AM Technological Merits and Nanogenerator Performance Optimization of Nanogenerators

**Fig. 7:**
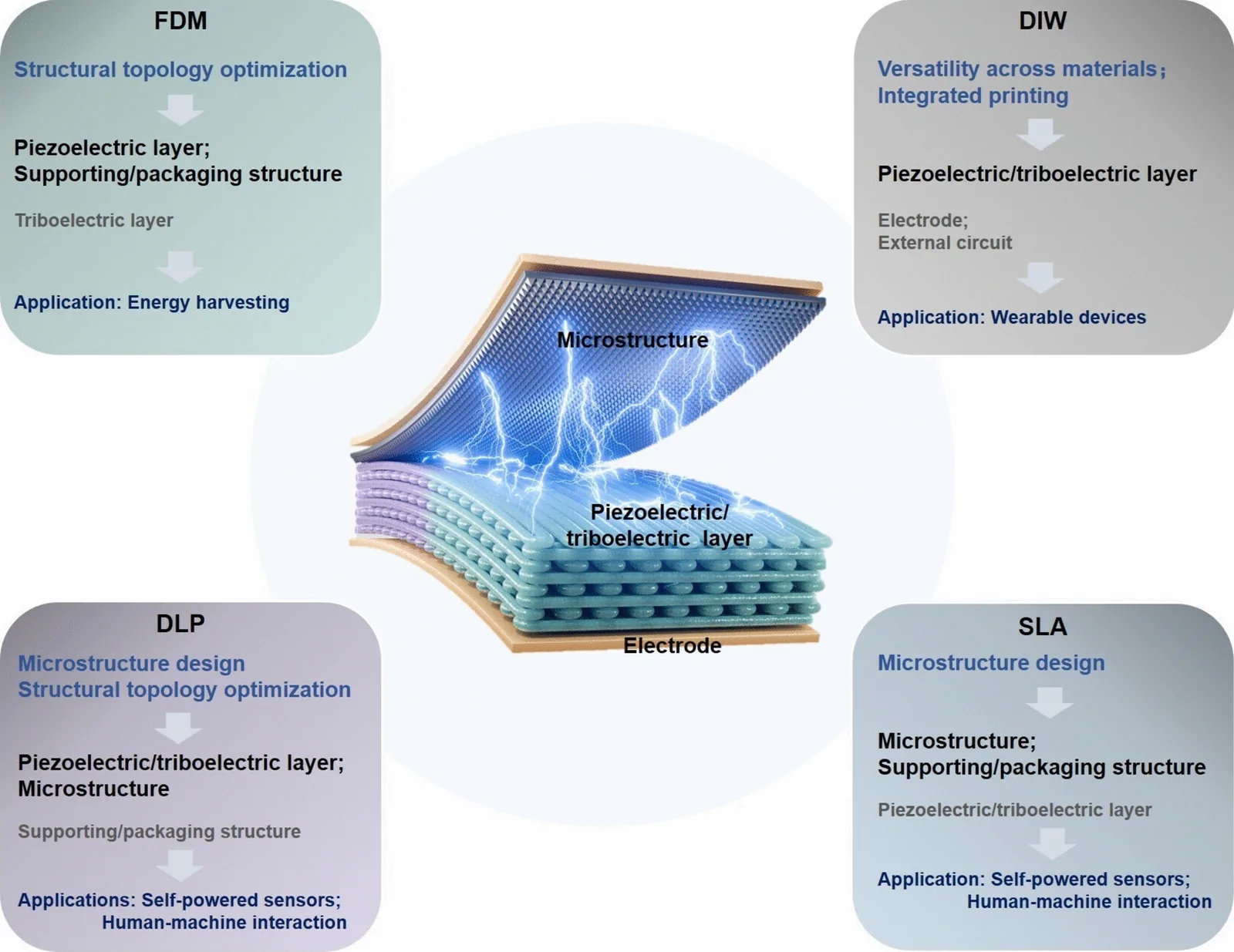
Hierarchical relationships connecting AM technological merits with nanogenerator optimization and applications

Based on the systematic analysis of the characteristics and recent advancements of commonly used AM technologies for nanogenerators, clear hierarchical relationships can be established to connect AM technological merits with performance optimization and applications of nanogenerators, as shown in Fig. 7. Section 5.1 systematically explores the merits of AM technologies in optimizing critical performance indicators of nanogenerators, as well as their suitability for fabricating different nanogenerator components. Section 5.2 primarily discusses the impact of AM processing parameter on the output performance of nanogenerators, offering valuable insights for practical implementation. The application scopes of high-performance nanogenerators enabled by different AM technologies are summarized in Sect. 5.3 from an application-oriented perspective.

### AM Technological Merits for Component Fabrication of Nanogenerators

In general, FDM technology is particularly well-suited for the structural topology optimization of nanogenerators, as exemplified by Fig. 3b(ii) and 3c(i). It is primarily used to regulate critical performance indicators of the devices, such as modulus, layer thickness, and interlayer gap distance, enabling the controlled reduction of stiffness, enhancement of deformation, and optimization of stress distribution, thereby significantly improving the output voltage, current, and power density of the nanogenerator. In addition, FDM is widely recognized for its cost-effectiveness, easy operation, and suitability for producing large, stable base structures. These attributes make it particularly advantageous for fabrication of supporting frames or packaging structures of PENGs/TENGs. By optimizing the framework, FDM effectively amplifies external mechanical stimuli, increasing their impact on the nanogenerator and consequently enhancing energy conversion efficiency [62]. The packaging structures offer encapsulation and protection to reduce charge dissipation and ensure output stability, as exemplified by Fig. 3e(i), while also enabling deployment in diverse environments for energy harvesting. It is worth mentioning that, compared to other AM technologies, FDM has some resolution limitations and is less commonly employed for fabricating ultra-precise microstructures in nanogenerators.

DIW technology exhibits outstanding versatility in processing a wide range of materials, alongside notable advantages in integrated printing, as exemplified by Fig. 4b(i) and d(i). This enables precise modulation of critical performance indicators of the integrated device, including the piezoelectric constant, surface charge density, and dielectric constant, thereby improving the overall output. These capabilities also facilitate the fabrication of all essential components of nanogenerators, including functional layers exhibiting piezoelectric or triboelectric properties, as well as electrodes, external circuitry, and supporting frameworks. Moreover, DIW is particularly effective in structural optimization of hierarchical or porous structures with precise control over porosity, as exemplified by Fig. 4a(i) and f(i). Research [183] has demonstrated that, within an appropriate range, increased porosity enhances the surface potential of nanogenerators, primarily due to elevated charge density. DIW-fabricated hierarchical or porous architectures significantly improve the tensile and compressive behaviors of the devices, thereby enabling exceptional mechanical responsiveness. Despite its advantages, DIW requires careful optimization of material rheological properties, such as ink viscosity and flow characteristics, and cannot reach the ultra-high resolution achievable with SLA and DLP technologies.

SLA technology excels in the fabrication of nanogenerators featuring intricate microstructures, complex geometries, and high-resolution surface patterns, as exemplified by Fig. 5a(i) and b(i). These features significantly increase the effective contact area and promote stress concentration, which in turn facilitate charge migration and accumulation, thereby enhancing the output performance of the device [184, 185]. SLA is also particularly well-suited for fabricating the internal supporting structures and external encapsulation structures of integrated and miniaturized nanogenerators, as exemplified by Fig. 5d(ii) and e(i). This helps mitigate charge neutralization and dissipation caused by external environmental factors such as humidity and dust, thereby enhancing the stability of the output. While SLA ensures ultra-high precision, its point-by-point scanning and curing process limits manufacturing efficiency, requiring post-curing under UV and removal of support structures.

DLP technology, similar to SLA, offers an ultra-high resolution, making it suitable for printing piezoelectric or triboelectric layers with a submicron or even nanometer-scale precision [186], as exemplified by Fig. 6d(i) and e(i). It can fabricate surface morphologies with uniformly arranged patterns and construct micro-/nanoscale contact interfaces, effectively increasing the material contact area to enhancing the charge migration [187]. Unlike SLA, DLP can cure an entire layer at once, significantly improving production efficiency. Furthermore, the superior ability of DLP to fabricate intricate ultra-precision structures facilitates fine-tuning of critical indicators of the devices, including modulus and piezoelectric constants in topology optimization, as exemplified by Fig. 6b(iii) and c(ii). However, the print size is limited by the optical system of DLP equipment, restricting its ability to fabricate large- sized nanogenerators in a single process. Notably, both DLP and SLA technologies generally require the material to be pre-treated into a photosensitive form, typically by incorporating photosensitive groups or adding photoinitiators.

### Impact of AM Processing Parameters on Nanogenerator Performance

For FDM and DIW, which are layer-by-layer manufacturing techniques based on material extrusion, certain specific process parameters—such as the printing path, printing speed, layer height, and nozzle temperature—can also influence the performance of nanogenerators to some extent. The printing paths can typically be categorized into two types: parallel and vertical. In parallel printing, the printing direction remains consistent across all layers, whereas in vertical printing, adjacent layers are printed in orthogonal directions. Nanogenerators fabricated using parallel printing generally exhibit slightly higher output than those produced with vertical printing, although the difference is not pronounced [188, 189]. This may be attributed to the lower stiffness of the parallel-printed device compared to the vertically printed one, despite having the same Young’s modulus of the material, thereby allowing greater deformation during compression. This increased deformation enhances polarization charge density and improves contact with the electrodes. On the other hand, nanogenerators with vertical printing tend to exhibit a slightly faster response time, due to the formation of intersecting fiber protrusions on the surface of the topmost layer, which facilitates quicker contact with the electrode or the opposing triboelectric layer. In the fabrication of nanogenerators, the printing speed directly affects the deposition pattern of materials and may have a potential impact on the arrangement of their molecular structures. A slower printing speed facilitates tighter adhesion between material layers, potentially resulting in higher charge density [190]. This effect is particularly pronounced when using viscoelastic materials, as slower speeds improve the consistency of molecular alignment, thereby promoting enhanced charge accumulation. Moreover, a reduced printing speed allows for a more orderly material arrangement during deposition, which may further promote the formation of the β-phase in piezoelectric materials such as PVDF, leading to improved piezoelectric response and output [191]. However, if the printing speed is excessively slow, it can cause issues such as stringing, where the material trails or extends in certain areas, negatively impacting surface quality [192]. Furthermore, overly slow speeds may result in excessive adhesion of the material to the print surface or nozzle, compromising printing accuracy. This can also impair material flow, leading to uneven adhesion and physical properties across different layers, thereby diminishing the mechanical response and overall performance of the nanogenerator. Additionally, layer height refers to the vertical thickness of each deposited material layer in FDM and DIW. Generally, a smaller layer height results in a smoother surface and higher printing resolution, although it also leads to increased printing time. A reduced layer height contributes to a more uniform internal structure, which, in turn, enhances the charge density during the operation of nanogenerators. Furthermore, it improves the interlayer adhesion, minimizing issues such as delamination, voids, or cracks, thereby enhancing the mechanical behavior and stability of the nanogenerators [193]. Factors such as the extrusion head temperature and the inner-to-outer diameter ratio of multi-material coaxial printing can potentially influence the output performance of nanogenerators. These factors are associated with the various choices of materials and the predefined structural design. However, the exceptional flexibility of AM technologies enables continuous optimization and iteration of printing parameters, allowing for the identification of the most optimal configurations.

Additionally, in SLA and DLP, exposure time refers to the duration for which each layer or point is illuminated by light source. This parameter determines the degree of photopolymerization of the photosensitive materials, thereby influencing the cross-link density and the microstructural fidelity of the printed object [177]. The exposure time significantly affects the performance of PENGs, primarily by altering the internal structural density of the material and the pathways for stress transmission. In general, prolonged exposure results in a higher crosslinking density, which increases the stiffness of the cured resin, thereby reducing the material deformability under external mechanical stimuli and compromising the piezoelectric output. In the case of TENGs, insufficient exposure may lead to incomplete curing of surface microstructures, such as micropillars or pyramids. This can cause structural collapse or deformation, reducing the effective contact area of triboelectric layers during contact–separation or sliding. Consequently, this limits electron transfer efficiency and charge accumulation, adversely affecting the overall triboelectric performance. Additionally, it has been reported that as exposure time increases, the dimensional precision in the X and Y directions initially improves and then decreases, while the dimensional precision in the Z direction remains stable [194]. This variation in dimensional accuracy can influence the topological optimization and microstructural fidelity of the nanogenerators, ultimately affecting final output performance. Moreover, the tensile behavior is typically influenced by the printing orientation and is linearly correlated with the orientation angle: the tensile performance decreases as the orientation angle increases [171, 195]. In contrast, the stiffness of the printed device is relatively insensitive to orientation. Besides, an increase in the orientation angle leads to weaker interlayer bonding, consequently hindering stress transfer and charge accumulation, and thus reducing the output performance of the nanogenerator. Furthermore, factors such as layer height, print spacing, and laser beam width may influence the final output of the devices, yet the inherent flexibility of AM allows for precise adjustments to effectively address these concerns.

### Applications of AM-Enabled High-Performance Nanogenerators

From an application-oriented perspective, although nanogenerators fabricated using different AM techniques have been widely applied in energy harvesting, self-powered sensors, wearable devices, and human–machine interaction, each technique exhibits distinct characteristics and application priorities. Therefore, the appropriate selection of AM techniques is critical to the further development and deployment of nanogenerators. In general, FDM-fabricated nanogenerators are typically used in energy harvesting applications, such as wave energy collection or powering LEDs. This is due to the well-designed supporting and packaging structures, along with the topologically optimized design achieved by FDM, all of which contribute to high energy conversion efficiency and operational stability. DIW-fabricated nanogenerators are commonly used in wearable devices for monitoring human motion or physiological signals [134]. This is due to DIW ability to process a broader range of stretchable materials, enhancing comfort during wear, as well as biocompatible materials that ensure prolonged skin contact without inducing allergic reactions or other adverse effects [196]. Nanogenerators fabricated by SLA or DLP are particularly well-suited for self-powered sensors and human–machine interaction. The high resolution inherent in SLA and DLP enables the creation of highly precise microstructures or surface patterns, significantly enhancing the sensitivity of the devices. This increased sensitivity is critical for detecting subtle or coupled signals, making SLA/DLP-based nanogenerators ideal for applications that require precise signal detection and effective interaction. Representative studies on commonly used AM technologies for nanogenerators and their applications are systematically summarized in Table 2.

**Table 2:** Representative studies on common AM technologies for nanogenerators

AM technologies	Nanogenerators (PENG/TENG)	Enhanced output performance	Applications	References
FDM	PENG	V_OC_ = 6.62 V, 5 times higher than the original; *I*_SC_ = 87.6 nA	Energy harvesting	[128]
		*V*_OC_ = 8.6 V; *I*_SC_ = 280 nA; 2 times higher than the original	Energy harvesting	[145]
		*V*_OC_ = 9.7 V	Wearable device	[123]
	TENG	*V*_OC_ = 98.2 V; *I*_SC_ = 13.7 μA	Energy harvesting	[158]
		*V*_OC_ = 306 V, 34 V higher than the original; *I*_SC_ = 6.14 mA; Power density 236.67 W/m^3;^ Energy conversion efficiency 74.4%	Energy harvesting	[161]
		Peak power density 185.4 W/(m^3^·Hz)	Self-powered sensor	[160]
DIW	PENG	*V*_OC_ = 80 V; *I*_SC_ = 25 μA; Power density 242 μW/cm^2^, 9 times higher than the original	Wearable device	[129]
		*V*_OC_ = 150 V; *I*_SC_ = 16 μA; Peak power density 64.8 μW/cm^2^	Wearable device	[125]
		*V*_OC_ = 6 V; Current density 2 μA /cm^2^; Peak power density 1.4 μW/cm^2^	Wearable device	[165]
	TENG	*V*_OC_ = 54.8 V, 39 V higher than the original; *I*_SC_ = 1.2 μA, 0.54 µA higher than the original	Wearable device; Energy harvesting	[134]
		Peak power density 10.98 W/m^3^; Transferred charge per volume 0.65 mC/m^3^	Wearable device	[135]
		*V*_OC_ = 60 V; *I*_SC_ = 0.23 μA; *Q*_SC_ = 58 nC; Peak power density 15.59 W/m^2^	Self-powered sensor; Human–machine interaction	[64]
		*V*_OC_ = 8 V; *I*_SC_ = 145 nA	Energy harvesting	[137]
SLA	PENG	Sensitivity 24 mV/kPa, 10 times higher than the original	Self-powered sensor	[66]
		*V*_OC_ = 10 V, 4 times higher than the original; Sensitivity 30 mV/kPa	Self-powered sensor	[127]
		*V*_OC_ = 385 mV; Piezoelectric charge constant 27.70 pC/N	Self-powered sensor	[172]
	TENG	*V*_OC_ = 10.9 ± 0.6 V; *I*_SC_ = 0.09 ± 0.02 μA	Self-powered sensor; Energy harvesting	[175]
		*V*_OC_ > 6 V;	Self-powered sensor	[176]
		*V*_OC_ = 4.5 V, 3 times higher than the original; Sensitivity 1.04 VkPa^−1^	Self-powered sensor	[185]
		*V*_OC_ = 21.8 V; *I*_SC_ = 0.25 μA; *Q*_SC_ = 6.4 nC	Energy harvesting	[65]
DLP	PENG	*V*_OC_ = 1.83 V, 8.3 times higher than original; Current density 82 nA /cm^2^	Self-powered sensor Wearable device	[67]
		Piezoelectric voltage constant 11.098 Vm/N; Normalized voltage 200 times higher than original	Human–machine interaction; Wearable device	[126]
		*V*_OC_ = 5.5 mV; *I*_SC_ = 5.2 pA	Energy harvesting	[187]
	TENG	*V*_OC_ = 201.4 V	Self-powered sensor	[131]
		*V*_OC_ = 1.15 V; *I*_SC_ = 6.61 μA	Self-powered sensor	[68]
		*V*_OC_ = 47.7 V; *I*_SC_ = 5.2 μA; *Q*_SC_ = 20 nC	Self-powered sensor; Human–machine interaction	[180]
		*V*_OC_ = 1.7 V/2.3 V; *Q*_SC_ = 5.4 nC/4.9 nC; 5 times/4 times than original	Energy harvesting	[133]

## Limitations and Challenges

**Fig. 8:**
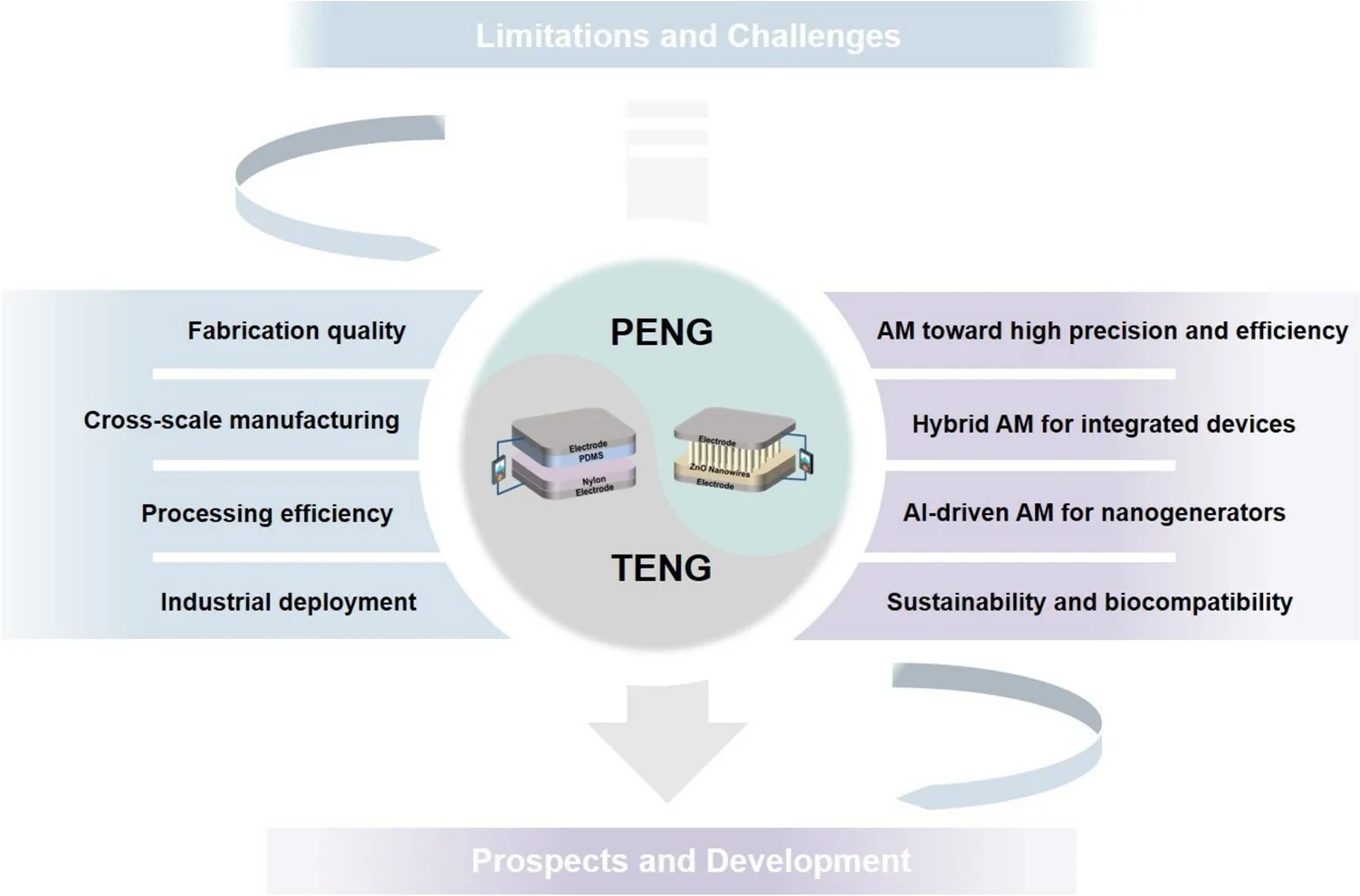
Limitations and prospects of AM for nanogenerators

Although AM has significantly contributed to the development of nanogenerators, enhancing their output performance and expanding their potential applications, inherent limitations and current challenges remain that need to be overcome (Fig. 8).

### Challenges of Fabrication Quality

AM for nanogenerator may face certain challenges related to fabrication quality, with the most prominent issues being surface roughness and interfacial bonding. In the fabrication of ultra-smooth and ultrathin film or fiber structures, AM, particularly techniques such as FDM, may result in increased surface roughness and thickness [197]. This is primarily due to resolution limitations and the influence of factors such as the printing path, printing speed, and nozzle temperature. Limited nozzle resolution may cause visible seams between deposition tracks, whereas suboptimal printing path scheme or non-uniform path distribution may induce localized waviness or step-like surface features [198]. In AM of thermoplastics, thermal stress and cooling rate during solidification influence surface roughness, while nozzle clogging, wear, or laser misalignment can also compromise surface quality and thickness uniformity. Appropriate post-processing strategies, including polishing, laser treatment, and other surface smoothing methods, can effectively reduce surface roughness without compromising the structural advantages inherent to AM [199, 200]. Additionally, AM-fabricated nanogenerators often incorporate diverse materials with distinct physical, chemical, and mechanical properties. These disparities can lead to variations in interfacial adhesion, particularly when there are pronounced differences in thermal expansion, surface energy, or chemical affinity [201]. These differences can result in inadequate interfacial adhesion, leading to delamination or peeling between the layers during integrated printing of AM [202]. Certain materials may undergo chemical reactions or diffusion at the melting or curing temperatures of adjacent layers, thus impacting interfacial bonding [203]. These challenges related to fabrication quality inevitably influence the performance of the nanogenerators.

### Limitations of Cross-Scale Manufacturing

The cross-scale manufacturing capabilities of AM for nanogenerators are still limited. The precision and uniformity of micro-/nanostructures are crucial for enhancing the output performance of the nanogenerators, thereby requiring AM technologies with a high resolution. However, many AM methods are not capable of providing a balance between the high precision and the flexibility required for fabricating large structures [204, 205]. Each type of AM technology typically specializes in a specific scale range. For instance, photopolymerization-based AM techniques, such as SLA and DLP, are highly effective at producing microscale structures with exceptional precision. While these techniques are limited in constructing large-area macrostructures due to constraints such as equipment size and light sources. In contrast, fused deposition-based AM techniques, such as FDM, are well-suited for manufacturing large- sized structures but lack the capability to achieve submicron or nanoscale resolutions. As a result, current AM technologies face practical limitations in consistently meeting the continuous manufacturing demands across a wide range of scales, from nanoscale to macroscale. These limitations hinder their ability to fully meet the forward-looking requirements of nanogenerators.

### Limitations of Processing Efficiency

Although AM offers a promising solution for the customized fabrication of complex nanogenerator structures, printing efficiency remains a major barrier to its widespread adoption, particularly in large-scale applications. Achieving high precision in AM typically requires finer nozzles or lasers, slower printing speeds, and lower material deposition rates, all of which can lead to reduction of overall production efficiency [206]. This issue is further exacerbated in ultra-high-resolution AM techniques, where point-by-point or layer-by-layer processing exponentially increases manufacturing cycle time. As a result, the substantial time and resource investments required for large-area or mass production of high-performance nanogenerators may not meet industrial-scale production demands. Improving printing efficiency and reducing manufacturing costs are essential for enabling the industrial adoption and commercial viability of the AM-based nanogenerators in practical applications.

### Challenges in Industrial Deployment

Current research on AM-based nanogenerators is predominantly carried out within controlled laboratory environments. However, most existing studies and evaluations have not adequately considered the complex and dynamic factors present in real-world environments [139]. In realistic deployment scenarios, nanogenerators are expected to function reliably and consistently over extended operational periods, often in environments that are far from ideal. They must endure mechanical fatigue, environmental aging, and unpredictable physical stresses. External factors—such as temperature fluctuations, high or low humidity levels, exposure to acidic or alkaline atmospheres, dust, and mechanical abrasion—can all degrade the materials or alter the surface charge behavior critical to nanogenerator operation. Additionally, internal factors such as long-term structural integrity, material delamination, electrode corrosion, or degradation of micro-/nano-patterned surfaces can lead to reduced efficiency or total device failure. Moreover, variations in resolution and fabrication precision among different AM systems make it difficult to ensure consistency across devices, thereby impacting the performance stability and reproducibility of nanogenerators during mass production [207]. Furthermore, although AM-based nanogenerators are progressing rapidly, the associated technologies and manufacturing standards remain underdeveloped, with an absence of unified industry protocols and standardized quality inspection procedures. This deficiency may result in compatibility issues among manufacturers, supply chains, and equipment during large-scale production and deployment, ultimately compromising device production and limiting the industrial application of nanogenerators [208].

## Conclusions and Future Prospects

In conclusion, AM offers substantial advantages of versatility across materials, structural topology optimization, microstructure design, and integrated printing for enhancing the output performance of nanogenerators and expanding their applications. Comprehensive comparisons between AM and conventional fabrication methods are conducted in terms of cost, efficiency, scalability, and sustainability. The connection of the advantages of AM and the optimization of critical performance indicators of nanogenerators is clearly established. Detailed quantitative analyses demonstrate that AM-fabricated nanogenerators exhibit substantial improvements in output metrics such as voltage, current, and power density. Moreover, the characteristics of commonly used AM techniques, including FDM, DIW, SLA, and DLP, are comprehensively summarized, offering valuable insights into the strategic selection of AM technologies for applications in energy harvesting, self-powered sensors, wearable devices, and human–machine interaction. Importantly, the hierarchical relationships connecting AM technological merits with nanogenerator optimization and applications are systematically explored. Despite notable progress in AM for nanogenerators, several limitations that hinder its development still warrant attention, including fabrication quality, cross-scale manufacturing, processing efficiency, and industrial deployment. To effectively address these challenges and meet the growing demands as well as future prospects for miniaturized integration, multifunctionality, wireless portability, and intelligence of AM-enabled nanogenerators, the following key research directions are proposed (Fig. 8):

### AM Advancement Toward High Precision and Efficiency

The development and implementation of advanced AM technologies with ultra-high precision represent the most fundamental and essential solution for addressing limitations in fabrication quality. For FDM and DIW, enhancements in printing resolution and efficiency can be achieved through the optimization of nozzle design. Shape-modulating nozzles, such as fixed, adaptive, and multi-output types, enable dynamic adjustment of the nozzle size and geometry to enhance printing resolution and speed [209]. For instance, the adaptive nozzle, by dynamically adjusting its outlet diameter through integrated actuators to actively modify the voxel size, effectively prevents instabilities such as under-extrusion and over-extrusion [210]. Property-modulation nozzles leverage mechanisms such as rotation, vibration, or external stimuli (e.g., magnetic fields or temperature) to dynamically adjust material properties in real time during extrusion [211, 212]. For example, magnetically responsive nozzles can align ferromagnetic particles, facilitating the in-situ fabrication of flexible devices with programmable deformation behavior [213, 214]. Multi-material nozzles support rapid material switching, mixing, and co-extrusion, thereby enabling the fabrication of functionally graded or heterogeneous structures while significantly improving overall manufacturing efficiency [215]. As an example, co-extrusion nozzles can simultaneously extrude both rigid and soft materials to construct lattice structures that combine high stiffness with enhanced toughness [216]. For SLA and DLP, precision and surface quality can be significantly improved through the fine-tuning of optical and process parameters [217]. Precise control over laser characteristics, including power, wavelength, and pulse duration, enables accurate regulation of the heating, melting, and cooling behavior of the photopolymer, thereby minimizing deformation caused by differences in thermal expansion coefficients and improving the dimensional accuracy of printed structures. Increasing the numerical aperture of the optical system can enhance spatial resolution and reduce edge-blurring effects [218]. In addition, multi-beam or beam-splitting systems allow for parallel processing, which not only significantly improves fabrication efficiency but also enables localized optimization of printing precision through the independent tuning of laser parameters [219].

Moreover, the optimization of printing paths and strategies is crucial for enhancing both fabrication precision and efficiency. From a software perspective, advancements in slicing algorithms can significantly mitigate error accumulation throughout the printing process. Notable examples include computed axial lithography slicing [220], curved layer slicing [221], and conformal slicing [222]. Adaptive layering methods also attract attention; these methods dynamically adjust layer thickness—applying thinner layers in regions with intricate features to achieve high precision, and thicker layers in simpler or larger areas to accelerate the process [223]. The widely used G-code programming in AM requires further refinement to address challenges such as interrupted printing paths and the lack of synchronization among multiple system modules. For instance, time-based synchronization approaches decouple the control of auxiliary devices from the G-code, enabling continuous printing paths and thereby minimizing the likelihood of defects or surface roughness during fabrication [147]. From a hardware perspective, high-precision motion control platforms may incorporate nanometer-scale linear guides, piezoelectric actuators, or air bearing stages to ensure accurate and stable positioning throughout the printing process. The control logic of stepper motors or pneumatic pumps can be further optimized to achieve precise volumetric flow regulation. Integrating sensors—such as those for displacement, temperature, and pressure—into a closed-loop feedback system facilitates real-time monitoring and dynamic regulation of critical printing parameters, including laser power, nozzle temperature, and curing time, thereby ensuring optimal fabrication quality [224]. Additionally, recent advances in submicron- and nanoscale AM techniques, such as two-photon polymerization (TPP) and micro-stereolithography (micro-SLA) [225–227], have enabled the fabrication of smoother surfaces and finer films or fibrous structures. For example, TPP, based on the two-photon absorption effect, enables nanoscale resolution, facilitating the ultra-precise fabrication of surface or internal micro-/nanostructures in nanogenerator components [228]. Furthermore, novel AM techniques can enable advanced gradient printing, facilitating the precise and gradual adjustment of specific physical or mechanical characteristics of nanogenerator materials in a 3D objective. This capability allows for the optimization of device performance and functionality across different regions. The advent of 4D printing technologies could provide the nanogenerators with greater adaptability and flexibility, enabling them to maintain optimal performance under dynamic environmental conditions [229].

### Hybrid AM for Integrated Devices

Hybrid AM systems combine multiple AM techniques to capitalize on their respective strengths, thereby addressing complex fabrication requirements. This approach effectively overcomes the inherent limitations of single AM technologies in cross-scale manufacturing and substantially improves overall processing efficiency [230]. Hybrid AM systems can incorporate various AM modules within a unified platform. Each module operates autonomously through a dedicated control system, because all the modules share a common working platform and transition between processes via instructions from an integrated control module [231]. For example, FDM can be employed for the production of complex support structures, DIW for the fabrication of triboelectric or piezoelectric layers, and SLA or DLP for the construction of surface microstructures. The hybrid AM systems impose elevated demands on intelligent control modules, automated operation modules, and high-precision positioning mechanisms. The intelligent control module employs digital programming to regulate the various AM processes and devices, facilitating the seamless transition between processes without disrupting the overall manufacturing workflow. Automated operation modules, such as robotic arms or conveyor belts, enable these transitions and operations while adjusting the process sequence based on predefined paths and strategies. Furthermore, high-precision positioning mechanisms are essential to ensure the accurate alignment of macro structures produced by FDM/DIW with the microstructures created by SLA/DLP. Additionally, Future nanogenerators could function as energy conversion devices, integrating complex sensing, feedback, and interactive capabilities. For example, Huang et al. employed AM to develop an intelligent cubic piezoelectric node (iCUPE), facilitating realization of a self-sustained artificial intelligence of things (AIoT) [232]. Such integrated devices are expected to be independently fabricated by advanced hybrid AM systems in the future, eliminating the need for manual assembly. Moreover, to promote the industrial application of integrated devices, future research needs to focus more on comprehensive performance evaluations under real-world conditions. This includes assessments of long-term stability, environmental robustness, and mechanical reliability. The development of protective packaging strategies, durable materials, and structural optimization designs aimed at enhancing durability is essential for bridging the gap between laboratory prototypes and field-deployable devices. Furthermore, establishing standardized manufacturing protocols, quality control procedures, and a compatibility framework is crucial to large-scale production and deployment of AM-enabled integrated devices, thereby promoting their industrial applications.

### AI-Driven AM for Nanogenerators

Artificial intelligence (AI)-driven AM has emerged as a prominent area of research [233]. AI can be employed to optimize various aspects of the AM process, including material selection, structural parameters, printing paths, and printing conditions. Machine learning and data analysis can intelligently and adaptively adjust the AM process, reducing limitations and errors in manual design while meeting complex design and performance demands [234]. Research [235] has demonstrated that the optimization of printing parameters through AI offers significant potential for minimizing surface irregularities. AI algorithms can systematically be used to determine the optimal combination of key parameters, including nozzle temperature, layer thickness, printing speed, nozzle diameter, and material density, thereby substantially improving surface quality. In addition, AI-assisted AM, through image recognition, enables real-time monitoring and analysis of data generated during the printing process, facilitating prediction and correction of deviations to ensure layer quality and structural integrity of the final printed structures. Moreover, the future of AI-assisted AM for the nanogenerators involves intelligent production, integrating IoT and big data technologies to enable end-to-end automation and management. Intelligent production systems will autonomously adjust process parameters, optimize production plans, and make real-time corrections in response to anomalies, minimizing human intervention and reducing downtime [236]. Finally, leveraging trained models, AI has significant potential to perform comprehensive evaluations of mass-produced nanogenerators fabricated via AM, ensuring their performance and durability during industrial deployment.

### Sustainability and Biocompatibility

Future developments in AM for nanogenerators must increasingly align with the overarching principles of sustainable development and environmental protection. Compared to conventional manufacturing methods, AM offers a practical pathway to reduce resource waste for nanogenerators due to its inherent layer-by-layer fabrication approach. This advantage can be further amplified by integrating advanced material recycling technologies within the AM process chain, enabling the reuse of feedstock materials and reducing the generation of manufacturing scrap [106]. Moreover, ongoing improvements in AM equipment design are expected to optimize energy efficiency, thereby lowering the overall energy consumption during fabrication. In parallel, the evolution of AM technologies facilitates the broader adoption of eco-friendly and biodegradable materials tailored specifically for nanogenerator applications. Beyond conventional materials, the integration of smart and self-healing materials into AM-fabricated nanogenerators presents a pivotal advancement [237]. These innovative materials possess the ability to autonomously repair damages or dynamically adapt their properties in response to environmental fluctuations. Such functionality not only prolongs the operational lifespan and reliability of nanogenerators but also significantly reduces maintenance requirements and associated resource expenditures over the device lifecycle. Due to the customization capabilities of AM, the nanogenerators also hold immense potential in bioengineering, such as electronic skin, implantable devices, and biosensors [238]. For these bioengineering applications, devices must exhibit exceptional biocompatibility to prevent rejection or adverse reactions when nanogenerators contact the human body for an extended period [204, 239]. Another promising application of the AM-based nanogenerators in bioengineering is regenerative medicine. Integrating AM-based nanogenerators with tissue engineering enables electrical stimulation to promote growth, repair, or regeneration of mammalian cells [137]. This approach presents novel opportunities to advance fields such as artificial tissues and organs.
